# 105 K Wide Room Temperature Spin Transition Memory Due to a Supramolecular Latch Mechanism

**DOI:** 10.1021/jacs.2c05417

**Published:** 2022-07-28

**Authors:** Maksym Seredyuk, Kateryna Znovjyak, Francisco Javier Valverde-Muñoz, Ivan da Silva, M. Carmen Muñoz, Yurii S. Moroz, José Antonio Real

**Affiliations:** † Instituto de Ciencia Molecular, Departamento de Química Inorgánica, Universidad de Valencia, 46980 Paterna, Valencia, Spain; ‡ Department of Chemistry, Taras Shevchenko National University of Kyiv, 64/13, Volodymyrska Street, 01601 Kyiv, Ukraine; § ISIS Neutron Facility, STFC Rutherford Appleton Laboratory, Chilton, Oxfordshire OX11 0QX, U.K.; ∥ Departamento de Fisíca Aplicada, Universitat Politècnica de València, Camino de Vera s/n, E-46022 Valencia, Spain; ⊥ Chemspace Ltd., Chervonotkatska Street 78, 02094 Kyiv, Ukraine; # ChemBio Center, Taras Shevchenko National University of Kyiv, 60, Volodymyrska Street, 01601 Kyiv, Ukraine

## Abstract

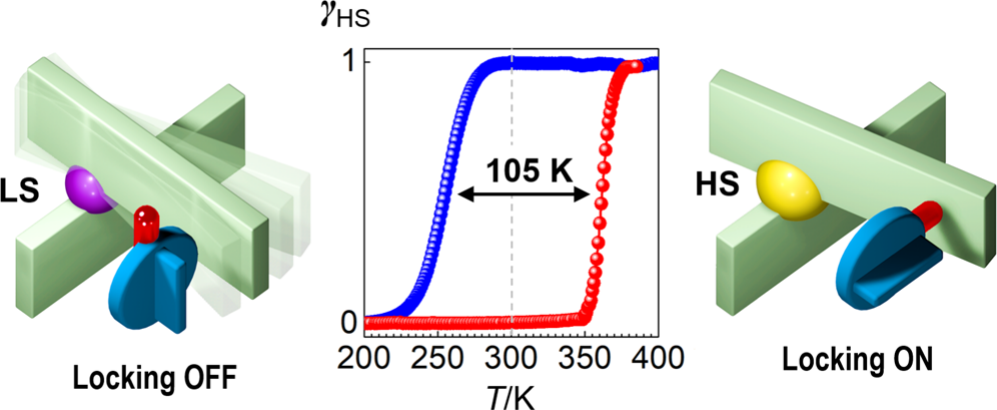

Little is known about the mechanisms behind the bistability (memory) of molecular spin transition compounds over broad temperature ranges (>100 K). To address this point, we report on a new discrete Fe^II^ neutral complex [Fe^II^L_2_]^0^ (**1**) based on a novel asymmetric tridentate ligand 2-(5-(3-methoxy-4*H*-1,2,4-triazol-3-yl)-6-(1*H*-pyrazol-1-yl))pyridine (L). Due to the asymmetric cone-shaped form, in the lattice, the formed complex molecules stack into a one-dimensional (1D) supramolecular chain. In the case of the rectangular supramolecular arrangement of chains in methanolates **1-A** and **1-B** (both orthorhombic, *Pbcn*) differing, respectively, by bent and extended spatial conformations of the 3-methoxy groups (3MeO), a moderate cooperativity is observed. In contrast, the hexagonal-like arrangement of supramolecular chains in polymorph **1-C** (monoclinic, **P**2_1_/*c*) results in steric coupling of the transforming complex species with the peripheral flipping 3MeO group. The group acts as a supramolecular latch, locking the huge geometric distortion of complex **1** and in turn the trigonal distortion of the central Fe^II^ ion in the high-spin state, thereby keeping it from the transition to the low-spin state over a large thermal range. Analysis of the crystal packing of **1-C** reveals significantly changing patterns of close intermolecular interactions on going between the phases substantiated by the energy framework analysis. The detected supramolecular mechanism leads to a record-setting robust 105 K wide hysteresis spanning the room temperature region and an atypically large *T*_LIESST_ relaxation value of 104 K of the photoexcited high-spin state. This work highlights a viable pathway toward a new generation of cleverly designed molecular memory materials.

## Introduction

The spin transition (ST) phenomenon is one of the most studied types of the molecular switching shortly celebrating a century of the unfading interest and fruitful discoveries, resulting in feasible expectations toward practical implementation into functional devices.^1−6^ The most appealing feature of Fe^II^-based ST materials is the bistability of physical properties (magnetic, optical, dielectric, *etc*.) due to the hysteretic transformation between the high-spin (HS, *S* = 2) and the low-spin states (LS, *S* = 0). The bistability was proposed for the information processing in nonvolatile memory bits,^2^ passive two-color displays,^2^ thermo/photomechanical actuators^7^ requiring a low energetic physical stimulus only at the moment of the spin state switching and further keeping the attained state form for an infinite time scale. The practical application of ST materials involves centering their bistability temperature range at room temperature (RT, 298 K), with the range itself overlapping or exceeding temperature variations, natural or caused by device self-heating, to exclude the temperature as a factor capable of accidentally initiating the ST, while the transition within the bistability region (hysteresis loop) can be accomplished by a selective physical stimulus such as light irradiation.^8−12^ In view of possible implementation into electronic devices, it is obvious that the ST bistability region must cover or exceed the adopted operating temperature range of electronic devices, which in the least strict case of consumer electronics embraces temperatures 0–70 °C (273–343 K, Δ*T* = 70 K).^13^ Among reported ST complexes, only a few have reproducible hysteresis width (Δ*T*_h_) ≥ 70 K wide,^14−17^ but so far for none the hysteresis overlaps with this temperature range. The lack of suitable bistable ST compounds is considered a fundamental obstacle to the practical application of this phenomenon in technological fields.

**Scheme 1:**
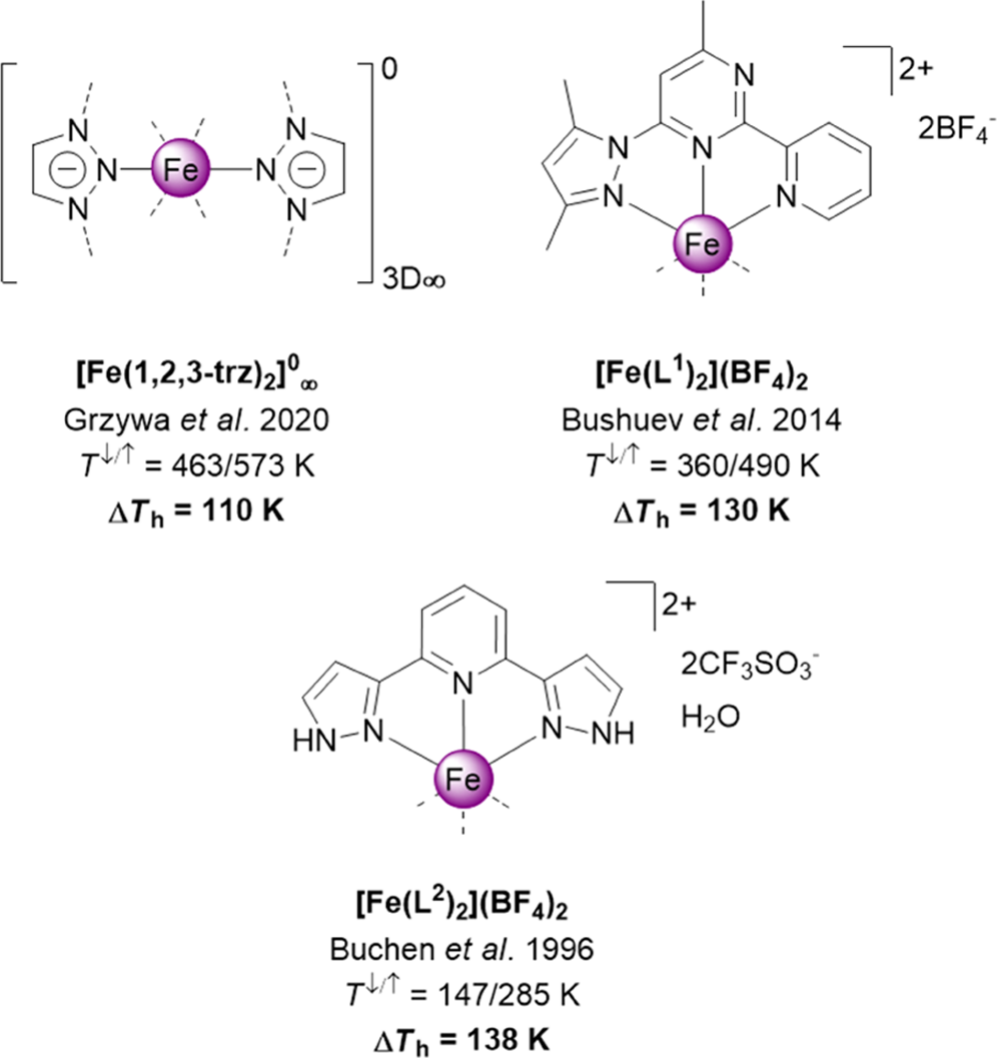
Literature Fe^II^ Complexes with a ST Hysteresis >100 K (L^1^ = 4-Methyl-2,6-bis(1*H*-pyrazol-1-yl)pyrimidine and L^2^ = 2,6-Bis(pyrazol-3-yl)pyridine)

The design of ST compounds with a specific transition temperature and especially hysteretic behavior remains a challenging task for synthetic chemists due to intrinsic difficulties associated with the control of supramolecular interactions in the solid state. While the ST temperature is mostly determined by the chemical nature of the ligand (by the ligand field strength), the hysteretic behavior is solely the result of the packing effects favoring, first, a synchronous spin-state change of the Fe^II^ centers,^18^ and, second, sufficient structural differences between the LS and HS phases as to create a thermal energy barrier responsible for the bistability.^19,20^ As for now, there is a knowledge gap on the structural mechanisms enabling bistability over large temperature ranges due to the absence of the structural data in both spin states for compounds with hysteresis loops exceeding 62 K.^21^ The only fully structurally characterized compound with the hysteresis >100 K is a 3D polymeric complex [Fe^II^(1,2,3-triazolate)_2_]^0^ (Δ*T*_h_ = 110 K), which undergoes ST with an outstandingly large and highly symmetric variation of the lattice (cubic → cubic).^17^ A detailed investigation of this unique compound attributed the origin of the behavior to the combination of a rigid lattice and ionic polarizable bonds due to the bridging triazolate ligands.^22^ For less symmetric discrete Fe^II^ compounds with meridional tridentate ligands exhibiting reproducible hysteresis exceeding 100 K^16,23,24^ (Scheme 1), the underlying structural mechanisms of the bistability remain unknown. Namely, the exceptional chemical variability of the tridentate ligands offers attractive prospects for tuning the transition temperature to the desired operating range.^25^

As suggested by the literature structural data of ST complexes in both spin states, realization of narrow hysteresis loops is usually associated with moderate structural differences between the LS and HS phases,^26−29^ whereas loops 20–60 K wide imply an accompanying lattice reorganization, including but not limited to order–disorder structural transitions of anions/solvent molecules/ligand substituents or lattice symmetry breaking.^16,21,30−34^ It can be assumed that similar mechanisms can be at the origin of hysteresis in numerous other ST compounds lacking structural characterization.^23,24,35−38^ In the case of complexes with tridentate ligands, these significant but hardly predictable lattice-level rearrangements can particularly lead to a trigonal distortion of the [FeN_6_] polyhedron.^39^ This kind of geometrical deformation is known to effectively decrease the ligand field strength by removing degeneracy and decreasing the splitting energy of the 3*d*-orbitals. Even, typically, LS Fe^II^ terpyridine-based complexes undergo a transition to the HS state due to a structural strain of the complex species induced by a phase transition of aliphatic counterions^40^ or field-induced trigonal twisting.^41^ The produced HS state of Fe^II^ ions could be kept as long as the external deforming forces are in action. Therefore, one can foresee that if the ST system would possess a built-in switching mechanism in the molecular periphery able to lock/unlock the transformation between a less distorted LS species and a strongly trigonally distorted HS species, this might be an excellent strategy to obtain compounds with wide hysteresis loops, thereby making a feasible design of bistable materials for the real-world applications.

With this idea in mind, and also having a positive experience with discrete systems exhibiting regulated ST due to significantly varying π–π interactions,^31,42,43^ we have focused our efforts on asymmetrically substituted neutral complexes with large planar ligands. The absence of nonactive “shock absorbers” of interactions such as counterions and solvent molecules and, instead, the numerous direct π–π and hydrogen intermolecular interactions between transforming ST molecules are considered important for the efficient spin-state transmission across the lattice.^23,44,45^ Furthermore, the asymmetric shape of the molecule is expected to segregate the packing into alternating regions with different interactions, which, besides favoring direct communication pathways between transforming Fe^II^ ions, can promote the cooperative transformation of the molecular periphery and thus increase the energy cost of the ST process.^31,42,43,46^

**Scheme 2:**
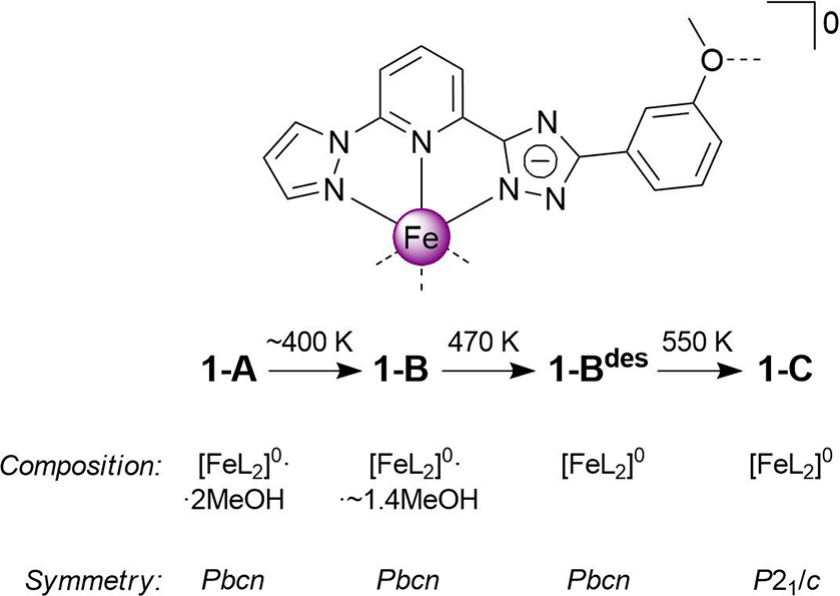
Schematic Molecular Structure, Transformation Path, Composition and Symmetry of the Title Compounds Two possible spatial orientations of the 3MeO group are shown: bent (solid Line) and extended (dashed line).

After several attempts, partially reflected in refs,^47−49^ we have concentrated our research studies on the ligand 2-(5-(3-methoxy-4*H*-1,2,4-triazol-3-yl)-6-(1*H*-pyrazol-1-yl))pyridine (L, see Scheme 2). Due to its asymmetric design, the neutral [FeL_2_]^0^ complex is a polar cone-shaped molecule favoring a one-dimensional (1D) stacking topology. Because of numerous but weak supramolecular interactions, the synthesized solvate compound [FeL_2_]^0^·2MeOH (**1-A**, *Pbcn*) is prone to polymorphic transformation and already after the first heating accompanied by gradual ST transforms into a partially desolvated polymorph [FeL_2_]^0^·∼1.4MeOH (**1-B**, *Pbcn*), exhibiting sharp ST without hysteresis near RT. Complete loss of the lattice methanol produces solventless phase **1-B**^**des**^ with a similar behavior but a higher transition temperature. Heating this complex above 550 K initiates an exothermic phase transition into the monoclinic polymorph [FeL_2_]^0^ (**1-C**, **P**2_1_/**c**), exhibiting ST with a hysteresis 105 K wide. Structural studies reveal a supramolecular latch mechanism involving one of the pendant 3MeO groups of the molecule. Depending on the spin state of the central Fe^II^ ion and the available intermolecular space, the 3MeO groups can adapt the bent or elongated conformations. The elongated conformation mechanically locks the trigonally distorted geometry of the neighboring molecule and thus favors the HS state of the central ion over a broad temperature range. In the bent conformation of the 3MeO group, the neighboring molecule has enough space to revert back to a less distorted geometry and convert the Fe^II^ ion to the LS state. Due to the spatial flipping of the 3MeO groups supported and stabilized by the concerted motion of the molecules favoring or disfavoring the trigonal distortion of the central metal ion, the studied compound **1-C** exhibits the most hysteretic ST behavior (memory) in the RT region known to date.

## Results and Discussion

### Synthesis

The ligand was conveniently synthesized by the Suzuki cross-coupling reaction of commercially available organic precursors (see the Supporting Information). Methanolate **1-A** was crystallized by layering a solution of the ligand and Fe(BF_4_)_2_·6H_2_O in acetone/chloroform (1:1) with a solution of triethylamine in methanol on the top. The formed cubic black crystals show good stability in air. Thermogravimetric analysis reveals that the full desolvation of the complexes occurs gradually in one step just above 400 K, whereas the desolvated complex is stable up to temperature above 600 K (Figure S1). According to the DSC data, the desolvation of **1-A** is accompanied by the ST (see below) and an additional structural phase transition with total Δ*H* = 129.5 kJ mol^–1^ of a broad asymmetric signal with a peak at 442 K. When heated to 400 K at 4 K/min, the partial solvate **1-B** can be isolated, and the fully desolvated phase **1-B**^**des**^ is obtained by direct heating to 470 K. Further heating of the compound up to *ca*. 550 K initiates an exothermic monotropic phase transition with Δ*H* = 11.9 kJ mol^–1^ into a new phase **1-C** (Figure S2).

### Magnetic and Calorimetric Studies

**Figure 1:**
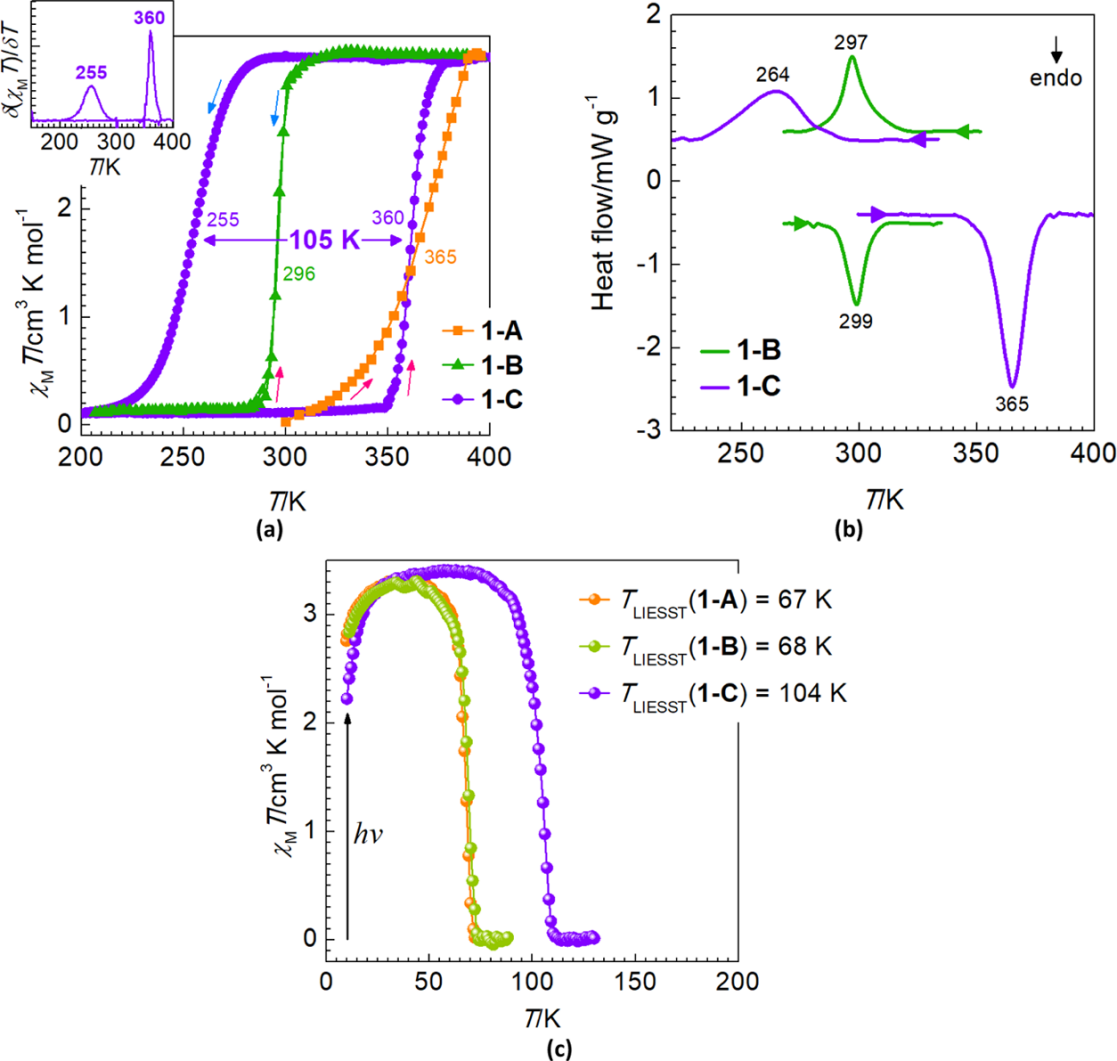
(a) Plot χ_M_*T* vs *T* for **1-A** and **1-B** at 1 K min^–1^ and **1-C** at 0.1 K min^–1^; (b) DSC profiles of the compounds at 10 K min^–1^; (c) LIESST curve of the compounds.

Magnetic susceptibility data were used to follow the changes of the ST behavior observed after polymorphic transformations. Initially, the magnetic measurements performed on a crushed polycrystalline sample of the as-synthesized **1-A** demonstrate its diamagnetic LS nature at RT (χ_M_*T* = 0) (Figure 1a). Upon heating, the sample undergoes an irreversible gradual ST with equilibrium temperature *T*_1/2_ = 365 K (at which the HS and LS fractions are equal to 0.5) and reaches a plateau with χ_M_*T* = 3.42 cm^3^ K mol^–1^ at 390–400 K.^50^ Cooling the sample back demonstrates a change of the magnetic properties related to the formation of a new phase **1-B**. The behavior of the new solvatomorph is characterized by an abrupt change of χ_M_*T* consistent with a complete ST upon cooling and then subsequent heating with the coinciding critical values *T*_1/2_^↓^ = *T*_1/2_^↑^ = 296 K, Δ*T*_h_ = 0 (Figure 1a). Despite the compound containing *ca*. 1.4 molecules of methanol per complex molecule (see below), the magnetic behavior is well repeatable upon cycling at temperatures below 320 K (Figure S3a). The differential scanning calorimetric (DSC) profile of **1-B** has a single peak at 299 K on heating and a single peak at 297 K on cooling (Figure 1b). The average enthalpy (Δ*H*) and entropy (Δ*S*) variation values are 7.6 kJ mol^–1^ and 25.6 J K^–1^ mol^–1^, respectively, and are characteristic for weakly cooperative Fe^II^ systems. The entropy value is higher than expected from electronic considerations (spin degeneracy only: ^1^A_1g_ → ^5^T_2g_ transition, *R*  ln(5) = 13.4 J K^–1^ mol^–1^). The remaining entropy variation (25.6–13.4 = 12.2 J K^–1^ mol^–1^) accounts for the crystal and molecular vibration modes involved in the ST process. The magnetic behavior of the fully desolvated compound **1-B**^**des**^ is similar to **1-B** (Figure S4). The difference arises from the location of the ST process in temperature, which is now shifted to 312 K, while the values Δ*H* and Δ*S* remain similar to those of **1-B**.

The exothermic phase transition at 550 K changes completely the ST behavior of the compound. Upon cooling from RT at 0.1 K min^–1^, **1-C** exhibits a drop of the susceptibility to zero with the critical temperature *T*_1/2_^↓^ = 255 K characteristic of the transition from the HS to the LS state. Upon subsequent heating, the susceptibility grows abruptly back to 3.42 cm^3^ K mol^–1^ at *T*_1/2_^↑^ = 360 K that envisages a complete ST back to the HS state (Figure 1a). The shaped 105 K wide hysteresis loop is slightly sensitive to the scan rate and is robust toward thermal cycling as demonstrated by the good reproducibility without fatigue (Figure S3b, inset). The latter is not surprising since there are no inclusions such as volatile solvents. The differential scanning calorimetric (DSC) profile of **1-C** has a peak at 264 K on cooling and at 365 K on heating (Figure 1b). The average enthalpy and entropy values equal to 13.9 kJ mol^–1^ and 44.9 J K^–1^ mol^–1^, respectively, suggest more significant structural transformations in comparison with **1-B**, a fact consistent with the structural data below. The fitting of the hysteresis loop using the Slichter–Drickamer model with entropy and enthalpy fixed to the experiential values yields the cooperativity parameter Γ = 9.1 kJ mol^–1^ (see the comparison of the experimental and fitted curves in Figure S5). To the best of our knowledge, it is the largest value among Fe^II^ ST compounds reported up to now.^16,51^

### Photomagnetic Studies

The light-induced excited spin-state trapping (LIESST) experiment^52^ was carried out at 10 K by irradiating microcrystalline samples of **1-A**, **1-B**, and **1-C** with a red laser (λ = 680 nm). Under these conditions, the signal reaches a plateau in *ca*. 30–40 min that corresponds to the fully populated metastable HS* state (Figure 1c). Subsequently, the laser was switched off and the temperature increased at a rate of 0.3 K min^–1^. The increase of χ_M_*T* in the range 10–40 K is attributed to the zero-field splitting of the HS* state, *S* = 2, whereas above this temperature range, the values reach the maximum of 3.30–3.40 cm^3^ K mol^–1^. In all three cases, the relaxation is a one-step process with characteristic *T*_LIESST_ temperatures^53^ 67 K (**1-A**), 68 K (**1-B**), and 104 K (**1-C**), determined from δ(χ_M_*T*)/δ*T*. For the latter, the thermal quenching experiment gives even a higher value of the relaxation *T*_TIESST_ equal to 118 K (Figure S6). It was demonstrated that a linear correlation between the ST equilibrium temperature *T*_1/2_ and *T*_LIESST_ holds generally for different types of Fe^II^ complexes. In particular, for structurally related Fe^II^ complexes with tridentate ligands, the two physical quantities may be related by the empirical formula: *T*_LIESST_ = *T*_0_ – 0.3*T*_1/2_, with *T*_0_ = 150 K.^5,54,55^ The three experimental points, plotted as the mean value ⟨*T*_1/2_⟩ *vsT*_LIESST_, fall above the line, whereas for **1-C**, the point is exceptionally high and approximates the line of polymeric Prussian blue analogues, whose behavior is governed not by pure ST but by the charge-transfer-induced ST effect^56^ (Figure S7). Currently, only one ST Fe^II^ complex with tridentate ligands is known to exhibit a similar high *T*_LIEEST_ value with *T*_1/2_ near RT.^57^ As discussed by Chastanet et al., high values of the *T*_LIESST_ for discrete compounds imply high trigonal distortion of the coordination sphere during the LS ↔ HS* transition that usually correlates with a high cooperativity of the ST process at higher temperatures.^5^ Both the hysteresis width and the *T*_LIESST_ values of **1-C** imply that the molecular packing is creating a large energetic barrier slowing down the relaxation and increasing the lifetime of the metastable HS* phase. As shown below, the packing of **1-C** indeed exhibits an unusual arrangement allowing for extraordinary flexibility in adapting one of the largest reversible molecular reorganizations during the LS → HS transition.

### Crystal Structures

Aiming at understanding the subjacent structural peculiarities leading to the progressive increase in cooperativity of the ST upon thermal treatment of the as-synthesized sample, we have performed a complete structural study of the three obtained solvatomorphs.

#### Structure of 1-A

**Figure 2:**
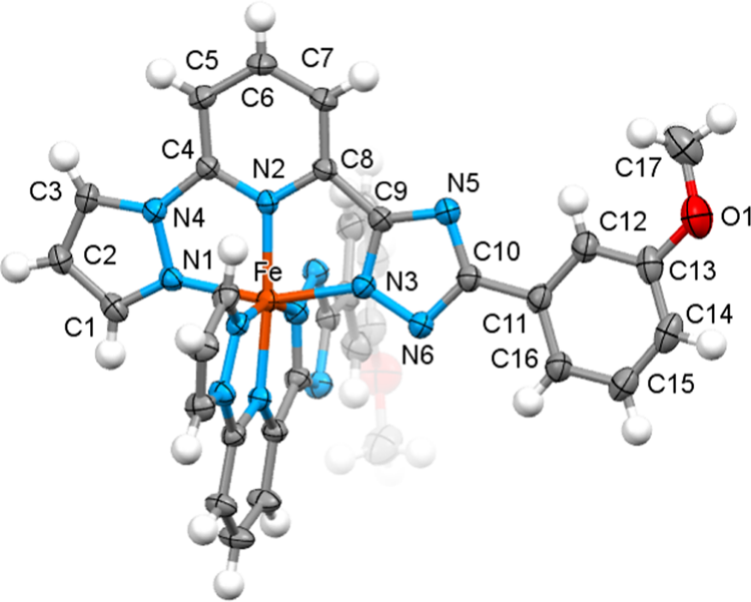
Molecular structure of **1-A** with the thermal ellipsoids at the 50% probability level. The methanol molecules are omitted for clarity.

Single crystals of **1-A** can be readily obtained by the slow diffusion method of an acetone/chloroform (1:1) solution of charged complex [FeL_2_](BF_4_)_2_ (bottom layer) and a methanol solution of NEt_3_ (top layer). Single-crystal X-ray diffraction (SCXRD) analysis reveals that complex **1-A** crystallizes in the orthorhombic space group *Pbcn* (see Table S1; for selected bond lengths and angles, see Table S2). The asymmetric unit comprises half of the complex molecule and a discrete MeOH molecule forming a hydrogen bond O(2)–H···N(5) with the triazole (trz) ring (Figure 2). The Fe^II^ ion has a pseudo-*O*_h_ coordination environment composed of the nitrogen donor atoms of pyrazole (pz), pyridine (py), and trz heterocycles with the averaged distance ⟨Fe–N⟩ = 1.946(4) Å (V^[FeN_6_]^= 9.509 Å^3^) consistent with the diamagnetic LS state of the complex. The trigonal distortion parameters are collected in Table 1. The ligand’s pz, py, trz, and ph rings as well as the 3MeO group essentially lay in the same plane. Furthermore, the 3MeO group is oriented toward the trz–py–pz ligand fragment, hereafter called bent configuration.

**Table 1:**
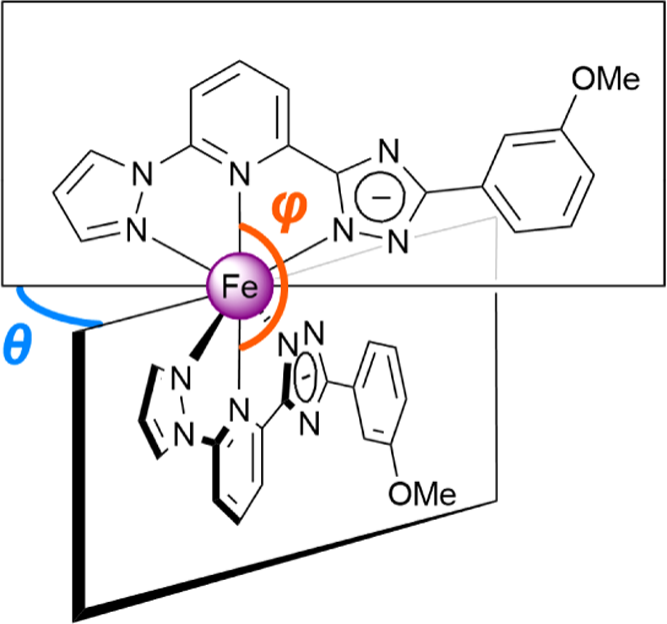
Distortion Parameters of the Coordination Polyhedron of **1-A**, **1-B**, and **1-C**^a^

	⟨Fe–N⟩, Å	Δ, Å	θ, °	Δθ	φ, °	Δφ
**1-A**(LS)	1.946		87.50		174.54	
**1-B**(LS)	1.95	0.19	87.55	0.78	178.28	–0.51
(HS)	2.14		86.77		178.79	
**1-C**(LS)	1.95	0.18	84.82	7.53	167.37	9.27
(HS)	2.13		77.29		158.10	

a The parameter θ is the dihedral angle (twisting), measured between the averaged planes of the fragments pz–py–trz, which is 90° in the case of the ideal *O*_h_ geometry of the coordination polyhedron [FeN_6_]. Parameter φ corresponds to the trans-angle N(py)–Fe–N(py′), which is 180° in the ideal case.

**Figure 3:**
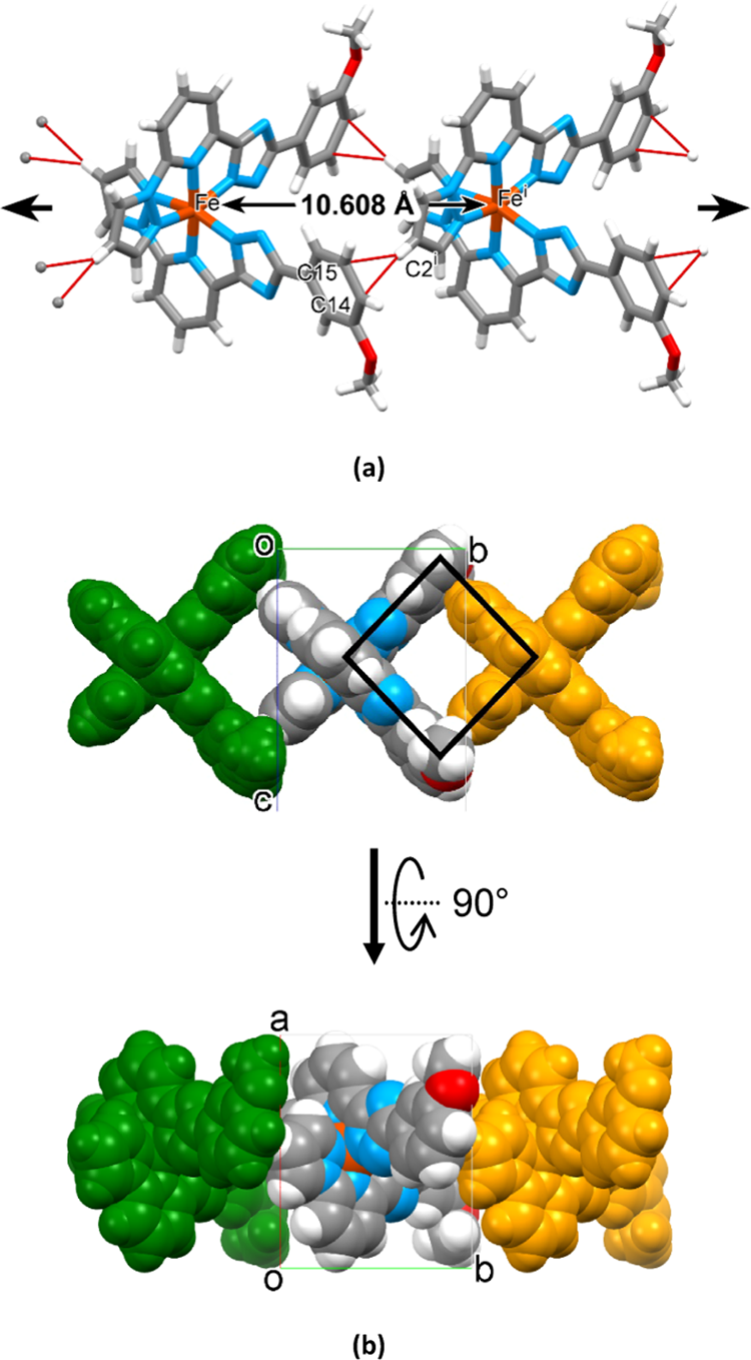
(a) 1D stack of molecules of **1-A** along axis *b* due to hydrogen bonding C–H···π(ph) (Symmetry code: (i) 1 – *x*, 1 + *y*, 1/2 – *z*). (b) Space-filling model of the stacking complex molecules illustrating the cavity between neighbor molecules within the stack, marked as a black rectangle.

The molecules of **1-A** have a conical shape with a smaller part (“head”) formed by two pz moieties and two longer divergent phenyl (ph) groups (“tails”) of the ligands with an Fe^II^ ion in between. The head of every molecule fits the cavity between the tails of the next neighbor molecule resembling the way the badminton birdies are stacked into columns. According to the Cambridge Structural Database (version 2021.3),^58^ this packing motif has never been reported for 3d complexes but is known for fullerene-based mesogens.^59,60^ Both pz moieties are oriented almost perpendicularly to the planes of the corresponding ph rings at a distance below the van der Waals (vdW) radii, forming the C–H···π bond {*d*[(pz)C2–H···C14*^i^*/C15*^i^*(ph)] = 2.857(8)/2.826(8) Å, *d*[(pz)C2–H···*C*_g_(ph)] = 2.771 Å; *i* = *x*, −1 + *y*, *z*; ∠[C–H···(plane of ph^i^)] = 77.083(3)°}. Hence, the formed infinite 1D column with the stacking periodicity 10.608(1) Å along axis *b* (= distance Fe···Fe = cell parameter *b*) also includes a rectangular void between neighboring molecules (Figure 3a,b). The electrostatic potential energy calculated using the B3LYP/6-31G(d,p) basis set localizes the negative charge on the trz–ph moieties of the complex molecule, while the pz–py moieties are relatively positively charged (Figure S8). The polar nature of the complex molecule justifies the realized 1D packing motif.

**Figure 4:**
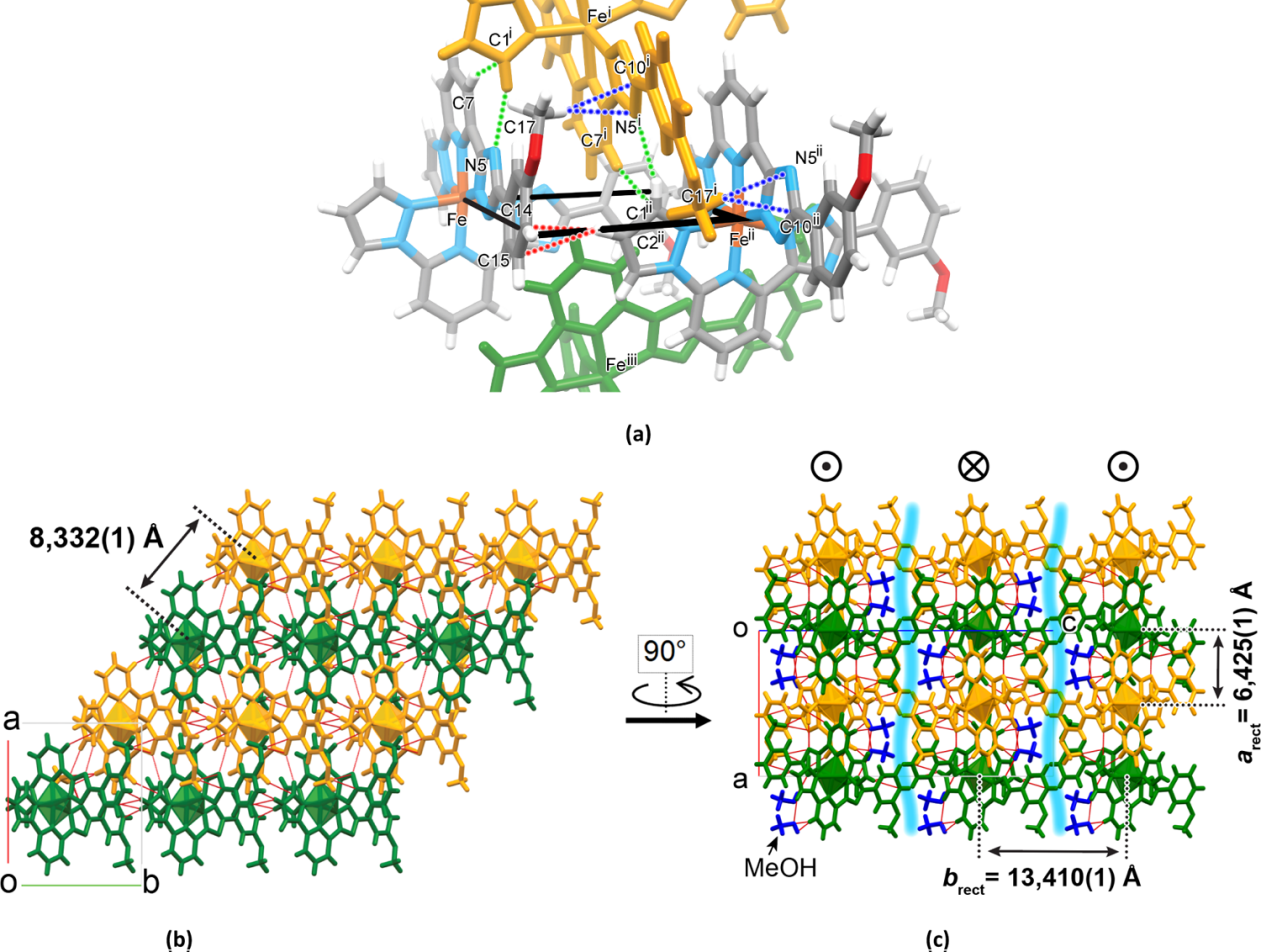
(a) Interactions below the vdW radii of the molecules from neighbor 1D stacks. For simplicity, only interactions with one neighbor molecule are shown. The black rectangle is the same as in Figure 3a. Symmetry codes: (i) 1 – *x*, 1 + *y*, 1/2 – *z*; (ii) −1/2 + *x*, 1/2 + *y*, 1/2 – *z*; (iii) 1/2 + *x*, 1/2 + *y*, 1/2 – *z*. (b) Stacks arranged in layers in the plane *ab.* Red lines correspond to the interactions below the vdW radii. (c) Layers stacking along axis *c*. The orientation of the molecules is changed to the opposite in neighbor layers. Blue shaded areas correspond to the interlayer space without intermolecular interactions below the vdW radii.

The molecules of neighboring columns tightly fit the void from both sides with the py moieties (Figure 4a). In addition to the abovementioned interaction, every pz moiety forms two weak hydrogen bonds (pz)C1–H···N5^*i*^(trz) and (pz)C1···H–C7^*i*^(py) with the intercalated planar ligands of the complex molecule from the neighbor column, and the same does the 3MeO groups, forming a double weak hydrogen bond C17–H···N5^*i*^/C10^*i*^(trz) (*i* = 1 – *x*, 1 + *y*, 1/2 – *z*). In this way, the 1D columns, shifted *b*/2 relatively to each other in the plane *ab* (Figure 4b), are joined defining slightly corrugated two-dimensional (2D) layers with the methanol molecules residing in the channels running along axis *b* (Figure 4c). At the highest level of packing complexity, the layers stack along axis *c*, although without any interactions below the vdW radii between the neighbor layers. Also, while within a layer, all molecules are oriented in the same direction, the orientation alternates in neighbor layers. The columns are packed in a rectangular way with the shortest intercolumnar distance *a*_rect_ = 6.425(1) Å and the longest *b*_rect_ = 13.410(1) Å (Figure 4c). The complete list of the intermolecular interactions is collected in Table S4.

#### Structure of 1-B

**Figure 5:**
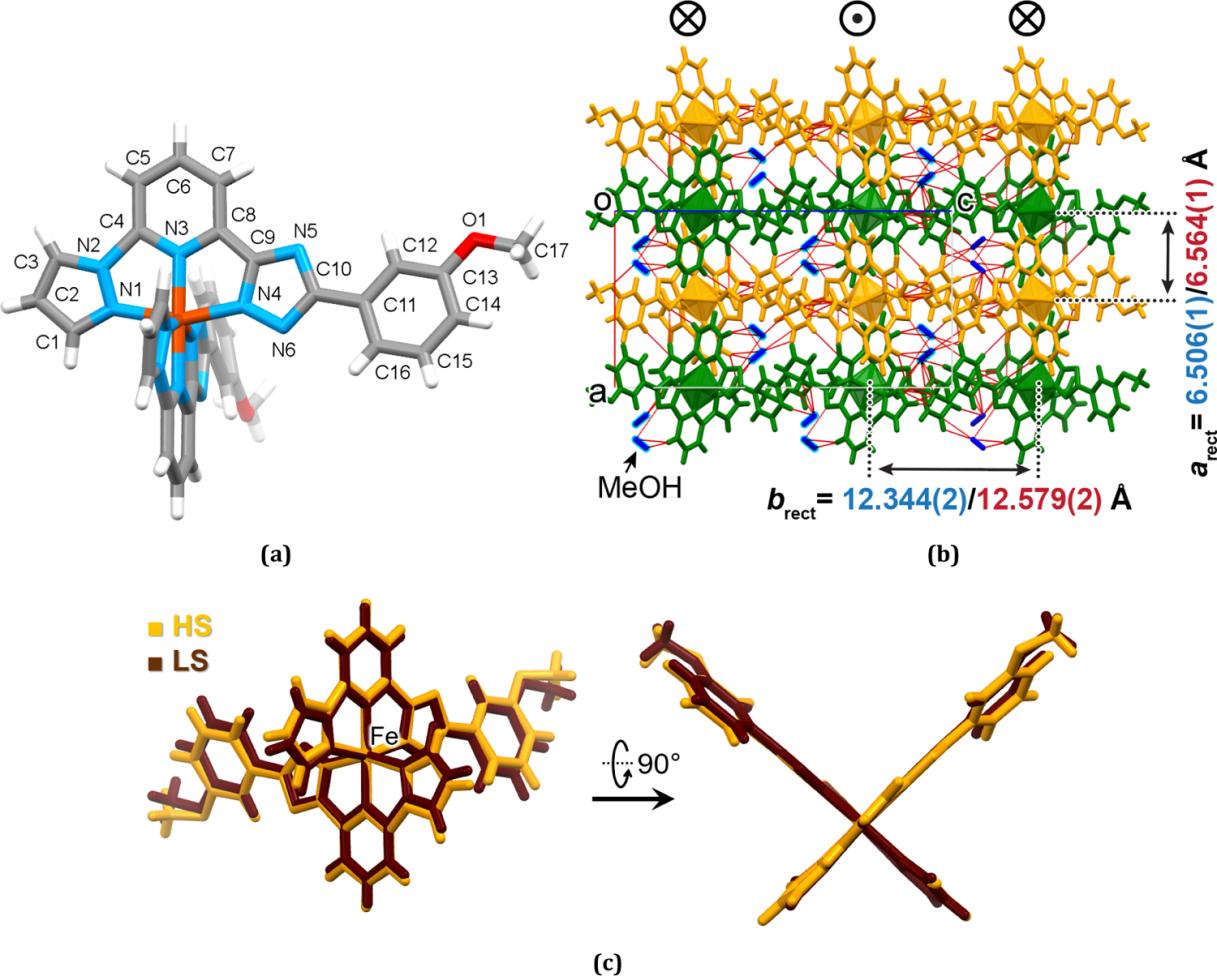
(a) Molecular structure of **1-B**. The methanol molecules are omitted for clarity and (b) projection of the 3D supramolecular arrangement with the rectangular packing motif of supramolecular chains with the interchain distances in the LS/HS states. Red lines correspond to the intermolecular contacts below the vdW radii. (c) Minimized overlay of the LS and HS complex molecules of **1-B**.

The partially desolvated phase **1-B** is characterized by an altered orientation of the pendant 3MeO groups and several resulting structural differences as revealed from the structural data in both spin states, which were refined by the Rietveld analysis (Figures 5a and S9). The lattice symmetry and the packing motif remain unchanged. The Fe···Fe separation within the supramolecular chains shortens slightly in the LS state compared to **1-A** (= 10.042(1) Å) and decreases further on going to the HS state (9.848(1) Å), the intercolumnar distance parameters *a*_rect_ and *b*_rect_ increase slightly (see Figure 5b). Similarly, while the *a* and *c* cell parameters increase slightly upon transition from the LS to the HS state (+0.9 and +1.9%, respectively), the *b* cell parameter decreases (−1.9%). This slightly anisotropic cell change is accompanied by a 0.8% increase in the cell volume (for details, see Table S3).

The major difference from the packing of **1-A** is engendered by the 3MeO groups, which are pointing now toward pz moieties of the molecules in the adjacent supramolecular layers: C17···C1*^i^*(pz), C17–H···C1*^i^*/C2*^i^*(pz), and C17–H···C10*^i^*(trz) (*i* = *x*, 1 – *y*, 1/2 + *z*) (see Table S5 for exact intermolecular distances), which unites stacks of supramolecular layers in a 3D supramolecular array. The number of short contacts below the vdW radii per molecule also increases but varies with the ST. The comparison of the LS and HS molecular structures demonstrates a change in the distance ⟨Fe–N⟩ from 1.952 Å up to 2.138 Å, respectively [Δ(⟨Fe–N⟩) = 0.186 Å], although the molecular shape remains essentially unchanged (Figure 5c). The corresponding trigonal distortion parameters are collected in Table 1.

Due to the fact that the PXRD profile of **1-B**^**des**^ is very similar to the corresponding profile of **1-B** (Figure S10), we can conclude that the local molecular and overall supramolecular arrangement is not affected by the complete desolvation and the lattice symmetry remains the same *Pbcn*.

#### Structure of 1-C

**Figure 6:**
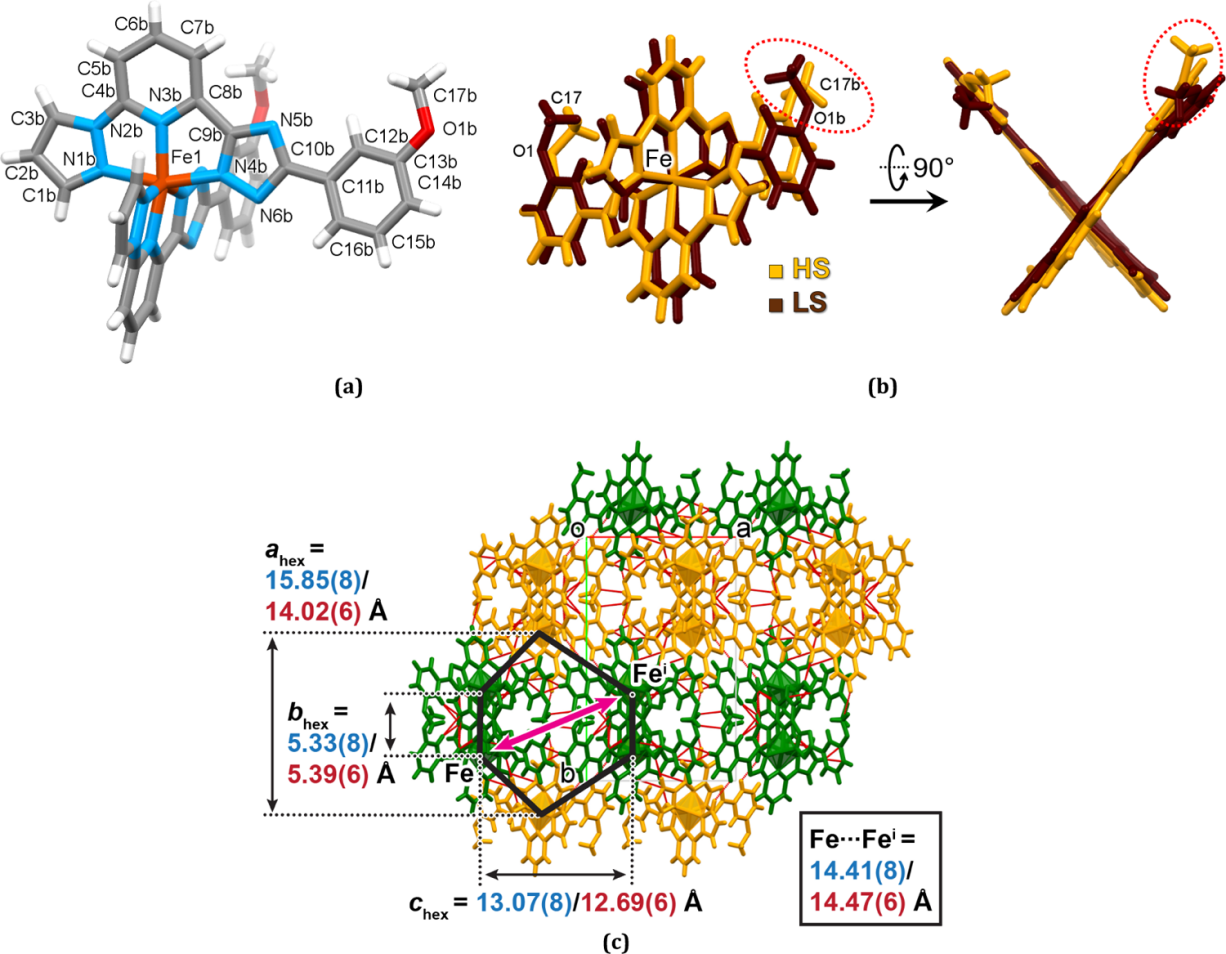
(a) Molecular structure of **1-C** in the LS states. For clarity, only the numeration of the ligand with the flipping 3MeO group is shown. (b) Overlay of the LS and HS molecules. Highlighted is the change of the conformation bent↔extended of the 3MeO group [−O(1b)C(17b)H_3_] due to the ST. (c) Projection of the crystal lattice along the *c* axis with the value of the *a*_hex_, *b*_hex_, and *c*_hex_ parameters in the LS/HS state. Red lines correspond to the intermolecular contacts. Symmetry code: *i* = −1 + *x*, 1/2 – *y*, 1/2 + *z*.

As a result of the phase transition of **1-B**^**des**^ at 550 K, the lattice symmetry decreases from orthorhombic (*Pbcn*) to monoclinic (**P**2_1_/**c**) with the significant change in the supramolecular arrangement of the crystal lattice. The structure of the resulting compound was refined from the high-quality X-ray diffraction data in both spin states at RT by the Rietveld analysis (Figures 6a and S11). It was found that the lattice exhibits a strongly anisotropic transformation due to the ST. While parameter *c* increases on going to the HS state (+11.0%), parameters *a* and *b* change by −1.9 and −8.4%, respectively. The total cell volume variation of only +0.1% is atypically low for an ST compound, which usually reaches 1–5%.^61^

Examination of the crystal structure of **1-C** in the LS state reveals the loss of the proper rotation axis of the molecules due to a change in the orientation of the 3MeO groups which have now the bent conformation, as in **1-A**, but are oriented in one direction (Figure 6a). In contrast, in the HS state, the 3MeO group [−O(1b)C(17b)H_3_] has the extended conformation similarly to **1-B** (Figure 6b). Changing the orientation of the 3MeO group, together with a slight change in the unit cell volume, results in a particular structural mechanism responsible for blocking the huge trigonal distortion of the molecule in the HS state and its retention over a broad temperature range. Before describing this revealed mechanism, let us first note that the packing of **1-C** preserves the 1D supramolecular arrangement but loses the layering. Unlike **1-A** and **1-B**, the py moieties enter deeper into the rectangular windows of the chain from one side, which prevents approaching of the chain by other py moieties from the opposite side. The two nearby chains, forming the double chain, are related by a glide plane and are connected by π–π stacking of large planar ligands and C–H···N interactions (Figure S12 and Table. S6). The outer half of the complex molecule, not involved in the formation of the double chains, participates in interchain interactions and has fewer contacts. The disparity of the supramolecular binding of the complex molecule in **1-C** causes the [FeN_6_] polyhedron to have a higher distortion in contrast to **1-A** or **1-B**. Already in the LS state, the angles φ and θ are lower than for the HS **1-B** and further decrease upon the ST to the HS state (Table 1).

The double chains pack in the *ab* plane in a honeycomb-like manner as shown in Figure 6c, characterized by arbitrary parameters *a*_hex_, *b*_hex_, and *c*_hex_. If the parameters *a*_hex_, and *c*_hex_ substantially decrease due to the ST, the *b*_hex_ remains almost unaffected. Similarly, the distance Fe···Fe^i^ for contacting molecules in the adjacent chains (see the red arrow in Figure 6c) remains practically unaltered [14.41(8) Å (LS)/14.47(6) Å (HS)]. Oppositely, the distance between the molecules within the chains increase from 11.442(7) Å (= cell parameter *c*) in the LS state up to 12.701(6) Å in the HS state.

To rationalize the anisotropic variation of the lattice, and particularly, the minor variation of the Fe···Fe^i^ distances of **1-C** on going between the spin states, which is essential in the observed behavior, we have performed the energy framework analysis^62^ in both spin-state phases. In this approach, the interaction energies between molecular pairs are represented as cylinders joining the centroids between two adjacent molecules, with the cylinder radius proportional to the magnitude of the interaction energy (the individual calculated electrostatic, dispersion, polarization, and repulsive contributions to the total interaction energy in both spin states using the B3LYP/6-31G(d,p) basis set are collected in Table S7). The interaction topologies of the molecules indicate the occurrence of strong cohesive interactions, drawn as thicker tubes on the total energy frame graphs (Figure S13). The difference framework^29^ identifies energetic changes across the LS → HS transition, which are mostly a stabilizing interaction (down to Δ*E* = −37.6 kJ mol^–1^), but also a prominent repulsing interaction of a similar amplitude (Δ*E* = 18.5 kJ mol^–1^) is observed (Figure S14 and Table S7). Interestingly, the difference framework of **1-B** also identifies a redistribution of interaction energies but with much lower amplitude in the range from −6.0 to +3.2 kJ mol^–1^, which correlates with the abrupt but nonhysteretic behavior of the compound (Table S8, Figures S15 and S16).^29^

**Figure 7:**
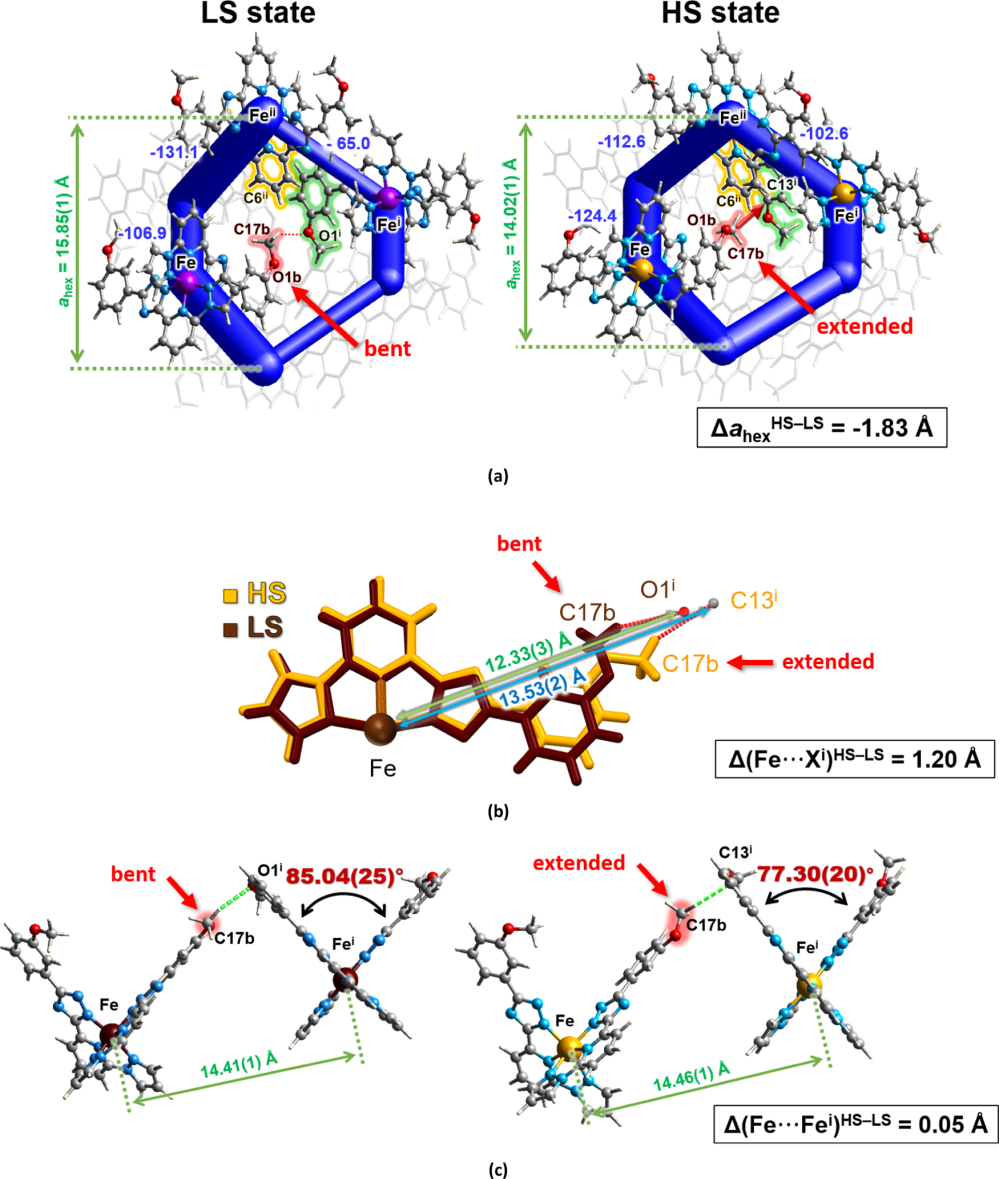
(a) Fragment of the energy frameworks merged with crystal packing of **1-C** in the LS phase in the *ab* plane (left) illustrating the change in the attractive interactions to a more uniform in the HS (right) and the resulting contraction eventually leading to the flip of the pendant 3MeO group (symmetry codes: (i) = −1 + *x*, 1/2 – *y*, 1/2 + *z*; (ii) = 1 – *x*, 1/2 + *y*, 1/2 – *z*). Tube size is 100, and cutoff is 50 kJ mol^–1^. The total interaction energy between pairs of molecules surrounding the hexagonal channel is indicated next to the corresponding tube. (b) Elongation of the ligand due to the flip of the 3MeO group from bent to the extended conformation. (c) Change of the interplanar angle *θ* due to the changed conformation of the 3MeO group while maintaining the distance between the molecules almost the same.

As can be inferred from the projection of the lattice of **1-C** in the *ab* plane (Figure 7a), the unequal strong attractive interactions between molecules in the LS state become more uniform in the HS state. This is a consequence of a better overlay of the planar ligand fragments and an overall significant change of intermolecular contact topology on going between the spin states (*cf*. changes for **1-B** and **1-C**, Tables S5 and S6, respectively), which increases the attraction forces and consequently tightens the packing, which compensates the increasing volume of the molecules due to the ST. The redistributed strength of the interactions explains a paradoxical decrease of the *a* and *b* cell parameters **1-C** that contradicts the expected 3D expansion of the lattice on going from the LS to the HS state.^17,63^ This, combined with an increase in the cell parameter *c*, results in some distances remaining intact, *e.g*. Fe···Fe^i^ in Figure 6c. Other distances evidently decrease as, for example, the ones characterized by the parameter *a*_hex_, which falls down by 1.83 Å across the transition to the HS state and which reflects the spatial convergence of the double chains to the center of the hexagonal channels. The resulting congestion of the organic moieties, particularly of the py moiety C(6)H and the 3MeO group [O(1b)C(17b)H_3_] highlighted in Figure 7a (d[C(6)–H···O(1b)] = 4.17(12)/2.79(8) Å in the LS/HS states), provokes the flip of the later from the bent conformation to the extended that elongates the ligand by 1.20 Å (Figure 7b). In turn, this group pushes the phenylmethoxy group of the contact molecule away and substantially decreases its both distortion angles φ and θ (Figure 7c) and increases the internal trigonal distortion of the [FeN_6_] octahedron to one of the highest values of Fe^II^ ST complexes with bisazolepyridine ligands.^21,39^ This strong geometric distortion is not observed for **1-B**; therefore, evidently, it is not a precondition for the ST of the molecule in **1-C**, but it is this structural factor that governs the hysteretic behavior of the compound. Importantly, the created structural hindrance is not irreversible, as is known from the experiment, but, nevertheless, the removal of the trigonal distortion by moving the 3MeO group sideways requires a great supercooling for the backward structural reorganization to occur. The same transformation pattern must be responsible for the exceptionally high *T*_LIESST_ value of **1-C** observed in the photoexcitation experiment described above.

**Figure 8:**
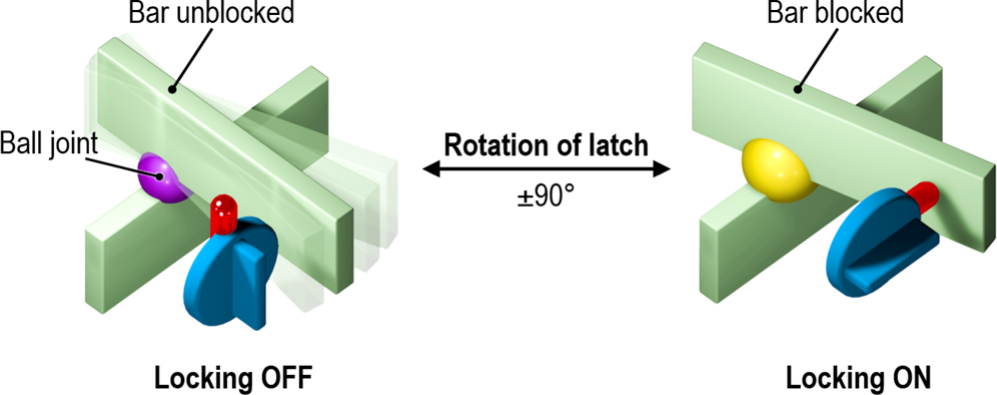
Schematic illustration of an open (left) and folded (right) scissor-like mechanism locked/unlocked by a rotary latch.

The mechanics of the geometric transformations that molecules of **1-C** exhibit when switching between the spin states is reminiscent of the way a rotary latch^64^ restricts the movement of an asymmetric scissor-like mechanism from the folded to the initial open configuration (Figure 8). In **1-C**, the role of the latch is played by the 3MeO group, flipping between two different spatial conformations stabilized by the nearest environment and available free space, while the planar ligands fastened by the Fe^II^ ion form a scissor-like mechanism. Here, the elongated ligand due to the extended 3MeO group mechanically blocks the return of the angles θ and φ values to higher values. To observe the backward motion of the scissor-like mechanism, the latch must first be raised. Similarly, in the case of **1-C**, the ligand length must be reduced by flipping the 3MeO group away from the plane of the phenyl moiety. Removing this mechanical obstacle makes room for the Fe^II^ molecule to restore a more regular geometry and to transit back to the LS state. In this mechanical model, the metal ion plays the role of a “ball joint” allowing both pivoting and tilt of the planar bars and corresponding distortion of the coordination polyhedron. It is important to note that for **1-C**, the rotation of the latching 3MeO group, the deformation of the molecule, the change of the distortion, and the ST of the Fe^II^ ion are occurring simultaneously in a highly cooperative concerted way that creates a large energetic barrier responsible for the observed huge hysteresis of the ST.

It is interesting to note that the known ST and non-ST molecular materials with robust thermal bistability in the range extending over 70 K near RT can be divided into two groups according to the amplitude of lattice structural rearrangements they undergo. Simple highly symmetric frameworks, such as charge-transfer Prussian blue analogues (Rb*_x_*Mn^II^[Fe^III^(CN)_6_]_(x+2)/3_·zH_2_O, Δ*T*_h_ = 86–138 K),^65,66^ the abovementioned 3D polymer [Fe^II^(1,2,3-triazolate)_2_]^0^ (Δ*T*_h_ = 110 K),^17^ simple small planar heterocyclic (1,3,5-trithia-2,4,6-triazapentalenyl, Δ*T*_h_ = 75 K),^67^ and metal–organic radicals ([Mn^II^(ADC^•^)(H_2_O)_2_ (DMF)_2_]_1D∞_, Δ*T*_h_ = 177 K)^68^ undergo structural hysteresis with relatively moderate lattice rearrangements, consisting in contraction/extension of the lattice and minor relative shifts/rotations/tilts of the lattice components. These are incomparable to the amplitude of the rearrangement of compounds with dynamic coordination environments, such as the bistable Ni^II^–porphyrin complex capable of switching the coordination environment of the Ni^II^ ion between planar N_4_ (*S* = 0) and square pyramidal N_5_ (*S* = 1), which requires sufficient free space for the grafted *cis-trans* photoisomerizable pendant group, ending with an N-donor pyridine fragment, to switch between the two conformations. Therefore, switching can be observed only in liquid media.^69^ In this sense, the hysteretic **1-C** occupies a niche between these two cases, combining an anisotropic “breathing” of the supramolecular structure that, at the same time, is flexible enough to adapt to the spatial flipping of the methoxy group.

**Figure 9:**
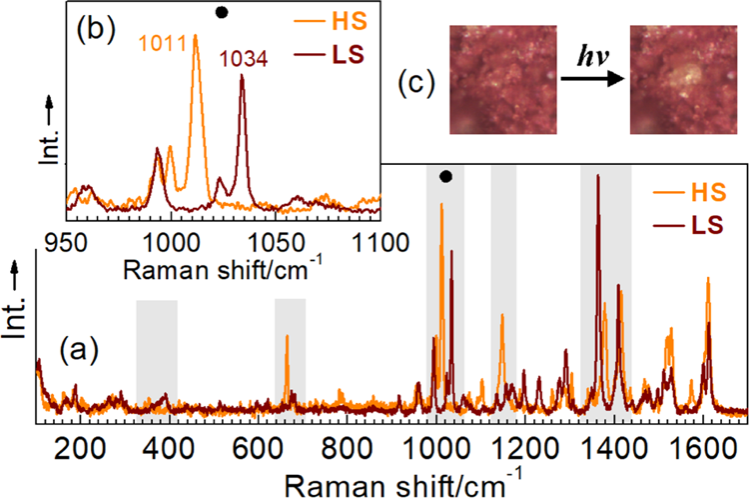
(a) LS and HS Raman spectra of **1-C**. Shaded areas indicate peaks with differences in position and intensity due to the ST. (b) Peaks correspond to the breathing vibration mode of the pyridine ring. (c) Yellow spot of the HS phase generated by the laser irradiation of the LS phase.

To extend the instrumental characterization of **1-C**, we have performed additional spectral characterizations of both spin-state phases coexisting at RT. Raman spectra of **1-C** compare well with those reported for Fe^II^ ST complexes with bispyrazolyl pyridine ligands.^21,70^ In the intraligand region 900–1700 cm^–1^, the LS and HS spectra differ in the position and intensity of the absorption bands (Figure 9a). Significant changes in the intensities of vibrations are observed upon the ST for bands centered around 1150, 1375, 1525, and 1735 cm^–1^. Clearly distinguishable is an intense characteristic peak at 1011 cm^–1^ in the HS state, belonging to a vibration mode of the pyridine ring, which shifts to 1034 cm^–1^ in the LS state due to the changed Fe–N bond length^71,72^ (Figure 9b). It is important to note that Raman scattering measurements are routinely made at 0.1 mW μm^–2^ laser power of the spectrometer (see the Supporting Information for details). By increasing the power up to 10 mW μm^–2^, it is possible to observe the quantitative conversion of the LS sample to the HS state during the experiment (Figure 9c). The Raman spectra of the thermally and photogenerated HS phases are indistinguishable (Figure S17). The UV–vis spectra of the solid-state sample apart from the intense transitions in the UV region due to the ligand show an MLCT band at 506 nm in both spin states (Figure S18). In the LS state, the pronounced brown coloration is due to an additional intense band centered at 560 nm, which disappears in the yellow-colored HS state.

**Figure 10:**
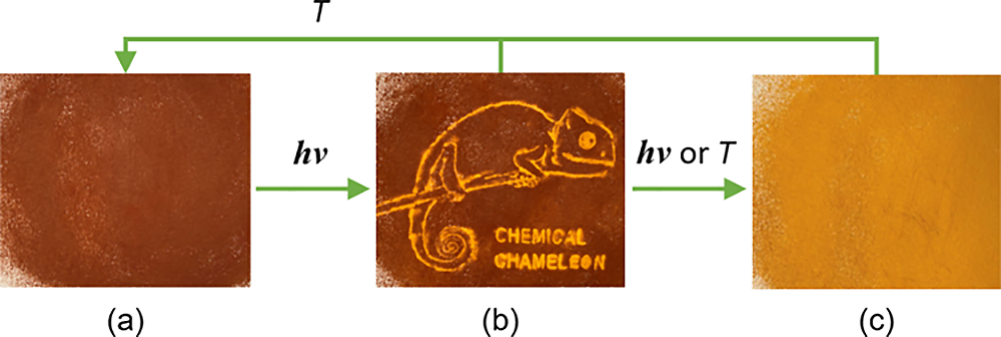
Irradiation of the LS **1-C** (brown) through a mask (a) with the formation of an image (b) and complete switching to the HS state (yellow) by further irradiation without mask or by heating (c). Reverse switching can be realized by cooling the sample below 250 K. The term “chemical chameleon” refers to the ability of the ST compounds, if bistable at RT, exist in differently colored states as a response to external stimuli.

The ability to change the spin states inside the ST bistability region by light is an attractive way for the contactless manipulation of physical properties of compounds.^8,73,74^ The Raman experiment and a pronounced thermochromism encouraged us to check the possibility to use **1-C** as a photosensitive ink for reproducing images (see the Supporting Information for details). In this regard, the irradiation of a macroscopic thin layer of the LS microcrystalline sample deposed on a filter paper through a mask with a laser diode (532 ± 10 nm, 10 mW) efficiently initiates the LS → HS switching. Due to the perfect bistability in the RT region, the resulting image is stable for months without any visible deterioration, it can be instantly blacked out by cooling below 250 K (Figure 10).

## Concluding Remarks

The present work describes the synthesis and characterization of an outstanding Fe^II^ compound of a new type, displaying an exceptionally wide hysteresis in the RT region and a high *T*_LIESST_ value. These properties have been achieved by a cleverly molecular design of the organic ligand favoring a singular supramolecular arrangement of the formed neutral metal complex and an unusual self-reproduced reorganization. Interestingly, there is no large change in entropy or enthalpy in the ST process, which is historically associated with highly cooperative ST processes. Instead, we demonstrate that the large trigonal distortion of the central metal ion, controlled by an inherent ON–OFF latch mechanism, traps the HS species and keeps them over a broad temperature range. Having clearly understood the way how the thermal bistability of ST can be programmed on purpose in the molecular structure, for example, by exploitation of the discovered supramolecular latch locking, we can foresee that new even more cooperative systems can be eventually obtained. This point is important because, from a general point of view, the technical implementation of an ST memory material is likely to require particle size reduction to the nanoscale, which is known to reduce coherent domains and to narrow the temperature range of bistability.^75^ Thus, systems with large hysteresis have a better chance of maintaining bistability when the particle size is reduced. The same principle applies to thin films. In this regard, obtaining thin films using a sublimation protocol may be an ideal method for nanostructuring the title complex compound, given its neutral nature and thermal stability, although, in this case, it is also important to preserve supramolecular structuring of **1-C** polymorph. Studies of cooperative systems and their nanostructuration are currently ongoing in our laboratories, and the results will be presented in due course.

## Supporting Information Available

Sample characterization, structural, spectroscopic, and computational data (PDF)
