# Supplementary material for: 105 K Wide Room Temperature Spin Transition Memory Due to a Supramolecular Latch Mechanism
Source: J Am Chem Soc. 2022 Jul 28;144(31):14297–309. doi: 10.1021/jacs.2c05417 (PMC9380689; doi:10.1021/jacs.2c05417)
Supplement: Supplementary file 1 — ja2c05417_si_001.pdf [file ja2c05417_si_001.pdf]

Supplementary Information

A 105 K wide room temperature spin transition memory due to supramolecular latch mechanism

Maksym Seredyuk*[ab], Kateryna Znovjyak[b], Francisco Javier Valverde-Muñoz[a], Ivan da Silva[c], M. Carmen Muñoz[d], Yurii S. Moroz[ef], José Antonio Real*[a]

[a] *Instituto de Ciencia Molecular/Departamento de Química Inorgánica, Universidad de Valencia, 46980 Paterna, Valencia, Spain. E-mail: maksym.seredyuk@uv.es; jose.a.real@uv.es*
[b] *Department of Chemistry, Taras Shevchenko National University of Kyiv, 64/13, Volodymyrska Street, 01601 Kyiv, Ukraine. E-mail: mcs@univ.kiev.ua*
[c] *ISIS Neutron Facility, STFC Rutherford Appleton Laboratory, Chilton, Oxfordshire OX11 0QX, UK*
[d] *Departamento de Fisíca Aplicada, Universitat Politècnica de València, Camino de Vera s/n, E-46022, Valencia, Spain*
[e] *Chemspace Ltd., Chervonotkatska Street 78, 02094 Kyiv, Ukraine*
[f] *ChemBio Center, Taras Shevchenko National University of Kyiv, 60, Volodymyrska Street, 01601 Kyiv, Ukraine*

CONTENTS

Materials: Synthesis of 2-(5-(3-methoxy-4*H*-1,2,4-triazol-3-yl)-6-(1*H*-pyrazol-1-yl)pyridine (L); Synthesis of the complexes. 2
Physical characterization. 2
Table S1. Crystallographic data for **1-A**. 4
Table S2. Selected bond length (Å) and angles (°) of the title compounds. 5
Table S3. Crystallographic data for **1-B** and **1-C**. 6
Table S4. Short intermolecular contacts of **1-A** below the van der Waals radii. 7
Table S5. Short intermolecular contacts of **1-B** below the van der Waals radii. 8
Table S6. Short intermolecular contacts of **1-C** below the van der Waals radii. 9
Table S7. Full color-coded interaction mappings of a central reference molecule with the nearest neighbors of **1-C** and the contributions to the total energy in both spin states as calculated by DTF-B3LYP/6-31G(d,p) method. 10
Table S8. Full color-coded interaction mappings of a central reference molecule with the nearest neighbors of **1-B** and the contributions to the total energy in both spin states as calculated by DTF-B3LYP/6-31G(d,p) method. 11
Figure S1. TGA profiles of the as-synthesized **1-A**. 12
Figure S2. DCS profile of the as-synthesized **1-A**. 12
Figure S3. Reproducibility of the ST behavior of **1-B** (a) and of **1-C** (b) at the rate 10 K min^{-1}. 12
Figure S4. Magnetic properties of **1-B^{des}** and DSC profile of fresh **1-A** 13
Figure S5. Fitting the magnetic curve of **1-C** by the Slichter-Drickamer model 13
Figure S6. Relaxation TIESST curve of **1-C** at 0.3 K min^{-1}. 14
Figure S7. Correlation T_{LIESST} vs. <$T_{1/2}$> for **1-A**, **1-B** and **1-C** 14
Figure S8. Electrostatic potential for the complex molecule of **1-A** derived from a B3LYP/6-31G(d,p) wavefunction mapped on the Hirshfeld surface in the range -0,1204 (red) to 0,1060 a.u. (blue). 15
Figure S9. Rietveld refinement plots for **1-B** in both spin states at indicated temperatures. 16
Figure S10. Comparison of the PXRD profiles of **1-B** and **1-B^{des}** 17
Figure S11. Rietveld refinement plots for **1-C** in both spin states at indicated temperature. 18
Figure S12. Intermolecular contacts of a double chain of **1-C** in the LS and HS spin states. 19
Figure S13. Coulomb and dispersion interaction energy components of the total energy frameworks of **1-C** in both spin states. 20
Figure S14. Differential framework of **1-C** constructed using values from the Table S7, column "Difference (LS–HS)", overlaid with a fragment of the crystal packing of the HS phase viewed along the *c* (a) and *b* (b) axes. 21
Figure S15. Coulomb and dispersion interaction energy components (above) of the total energy frameworks (below) of **1-B** in both spin states. 22
Figure S16. Differential framework of **1-B** constructed using values from Table S8, column "Difference (LS–HS)", overlaid with a fragment of the crystal packing of the HS phase viewed along the *a* axis. 23
Figure S17. Comparison of Raman spectra of the HS phase of **1-C** generated thermally and by laser irradiation of the LS phase. 24
Figure S18. UV-vis spectra of the LS and the HS phases of **1-C** at RT. 24
Photoirradiation experiment with **1-C** at RT 25
References. 26

Materials. All chemicals were purchased from commercial suppliers and used without further purification (Merck, Enamine Ltd.).

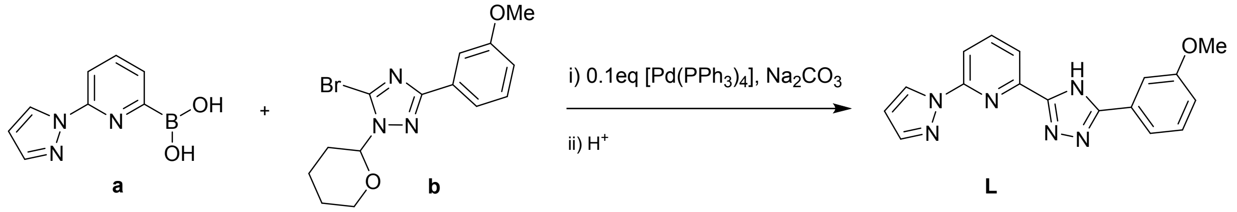

Synthesis of 2-(5-(3-methoxy-4*H*-1,2,4-triazol-3-yl)-6-(1*H*-pyrazol-1-yl)pyridine (L). A Schlenk flask with inert atmosphere was charged with 6-(1*H*-pyrazol-1-yl)pyridin-2-ylboronic acid (**a**, 1.00 g, 5.3 mmol), 5-bromo-3-(3-methoxyphenyl)-1-(tetrahydro-2*H*-pyran-2-yl)-1*H*-1,2,4-triazole (**b**, 1.63 g, 4.8 mmol), $[Pd(PPh_3)_4]$ (0.61 g, 0.53 mmol) and Na_2CO_3 (1.65 g, 15.6 mmol). Degassed 1,4-dioxane (20 mL) and degassed water (10 mL) were added, and the reaction mixture was heated to 100 °C under vigorous stirring for 16 h. After filtering through a Celite pad, to the obtained solution 5 ml of HCl_{aq} (37%) were added dropwise and the obtained solution was stirred for 10 min. Thereafter the pH of solution was brought to neutral with an aqueous solution of NaOH (10%). The resulting suspension was evaporated to dryness and resuspended in water, the precipitate was collected by filtration, washed with water and recrystallized from chloroform-acetone (1:1). The final compound was obtained after drying *in vacuo* as a white crystalline powder. Yield: 0.99 g, 65%. Elemental analysis calcd. for $C_{17}H_{14}N_6O$: C, 64.14; H, 4.43; N, 26.40. Found: C, 64.51; H, 4.26; N, 26.12. ^1H NMR (300 MHz, 25 °C, DMSO-d^6): δ (ppm) 14.90 (1H, br, trzH), 9.11 (1H, s br, pzH), 8.07 (3H, m, phH), 7.86 (1H, s, pzH), 7.74 (1H, dt, ^1J = 1.4 Hz, ^2J = 8.2 Hz, phH), 7.68 (1H, dd, ^1J = 1.4 Hz, ^2J = 2.6 Hz, phH), 7.42 (1H, t, J = 8.2 Hz), 7.01 (1H, ddd, ^1J = 1.4 Hz, ^2J = 2.6 Hz, ^3J = 8.2 Hz, phH), 6.64 (1H, dd, ^1J = 1.7 Hz, ^2J = 2.6 Hz, pzH), 3.85 (1H, s, CH_3). ^{13}C NMR (100 MHz, $CDCl_3$): δ 161.71 (br), 160.00, 155.05 (br), 151.03, 145.46 (br), 143.02, 141.42, 132.41 (br), 130.39, 128.58, 118.86, 118.75, 115.52, 113.02, 111.60, 108.73, 55.59.

Synthesis of the complexes

1-A ($[Fe(L_2)]^0\cdot 2MeOH$) was produced by layering in standard test tube. The layering sequence was as follows: the bottom layer contains a solution of $[Fe(L_2)](BF_4)_2$ prepared by dissolving L (100 mg, 0.314 mmol) and $Fe(BF_4)_2\cdot 6H_2O$ (53 mg, 0.157 mmol) in boiling acetone, to which chloroform (5 ml) was then added. The middle layer was a methanol-chloroform mixture (1:10) (10 ml) which was covered by a layer of methanol (10 ml), to which 100 µl of NEt_3 was added dropwise. The tube was sealed, and black cubic single crystals appeared in 3−4 weeks (yield *ca*. 60%). Elemental analysis calcd. for $C_{36}H_{34}FeN_{12}O_4$: C, 57.30; H, 4.54; N, 22.27. Found: C, 57.43; H, 4.52; N, 22.01.

1-B ($[Fe(L_2)]^0\cdot$~1.4MeOH) was prepared *in situ* by heating the sample of **1-A** inside the SQUID facility up to 400 K at 4 K/min. The obtained sample was used for the magnetic, DSC and PXRD characterizations. Elemental analysis calcd. for $C_{35.4}H_{31.6}FeN_{12}O_{3.4}$: C, 57.82; H, 4.33; N, 22.86. Found: C, 57.57; H, 4.60; N, 22.43.

1-B^{des} ($[Fe(L_2)]^0$) was prepared by heating **1-A** up to 470 K. Elemental analysis calcd. for $C_{34}H_{26}FeN_{12}O_2$: C, 59.14; H, 3.80; N, 24.34. Found: C, 58.98; H, 3.58; N, 24.74.

1-C ($[Fe(L_2)]^0$) was prepared by heating of **1-A** up to 600 K and annealing at 460 K for 72 h in Ar. Elemental analysis calcd. for $C_{34}H_{26}FeN_{12}O_2$: C, 59.14; H, 3.80; N, 24.34. Found: C, 58.76; H, 3.68; N, 24.77.

Physical characterization

Variable-temperature **magnetic susceptibility data** (15-20 mg) were recorded on samples at variable rates between 10-400 K using a Quantum Design MPMS2 SQUID susceptometer operating at 1 T magnet. The LIESST experiments were performed at 10 K in a commercial sample holder (Quantum Design Fiber Optic Sample Holder), wherein a quartz bucket containing *ca*. 1 mg of a sample was held against the end of a quartz fiber coupled with a red laser (633 nm, 15 mW cm^{-1}).

After reaching the saturation of susceptibility, the sample was heated up at the rate 0.3 K min^{-1}. The raw data were corrected for a paramagnetic background arising from the sample holder. The resulting magnetic signal was calibrated by scaling to match values with those of bulk sample. **Differential scanning calorimetric (DSC)** measurements were performed on a Mettler Toledo TGA/SDTA 821e under a nitrogen atmosphere with a rate of 10 K min^{-1}. The raw data were analyzed with the Netzsch Proteus software with an overall accuracy of 0.2 K in the temperature and 2 % in the heat flow. **Thermogravimetric analysis (TGA)** was performed on a Mettler Toledo TGA/SDTA 851e instrument, in the 290–1200 K temperature range under a nitrogen atmosphere at a rate of 10 K min^{-1}. **Elemental CHN analysis** was performed after combustion at 850 °C using IR detection and gravimetry by means of a Perkin–Elmer 2400 series II device. **NMR** spectroscopic measurements were done on an Advance DRX Bruker 300 MHz Spectrometer. **Raman spectroscopy:** The Raman spectra of **1-C** in the HS, LS and mixed HS/LS were recorded at 293 K using a HORIBA LabRAM HR Evolution equipped with a 633 nm Laser beam with a maximum adjustable power of 10 mW µm^{-2} and a 50× lens to focus the beam. The bulk material was placed directly on a glass slide and measured. All spectra, collected between 100 cm^{-1} and 1800 cm^{-1}, were normalized to facilitate their comparison. The pure LS state spectrum was recorded at 1% of maximum power for 10 sec. No spot stemming from irradiation in these conditions was observed on the surface of the sample. In contrast, a clear yellowish spot appears after irradiating the LS sample for 1 min at 100% of power, then the spectrum of so photogenerated HS state was recorded at 1% of power for 10 s. **UV-vis:** UV-vis spectra was recorded at room temperature for the LS and HS spin state solid phases of **1-C** using Jasco V-670 UV/Vis/NIR spectrophotometer. **Powder X-ray diffraction** measurements where performed on a PANalytical Empyrean X-ray powder diffractometer (monochromatic Cu K_{α} radiation) equipped with a PIXcel detector operating at 40 mA and 45 kV. PXRD data for **1-B** and **1-C** for Rietveld refinement were collected in the 5–90° (2θ) angular range with a step size of 0.013° and using a 0.5 mm glass capillary and soller slits of 0.02° and a divergence slit of 1/4°. DICVOL06 was used to determine the cell parameters by indexing the PXRD pattern of **1-B** and **1-C** in both spin states. The yielded unit cell parameters were further refined against the experimental PXRD in the corresponding space group by the LeBail method using the FULLPROF software package. These results were used to build a structural model of the compounds with Materials Studio (MS) 2017 based on the original structure of **1-A**, solved by SCXR measurements. This model was used as a starting point for a Rietveld refinement, which was carried out with TOPAS Academic 5 program (http://www.topas-academic.net/). **Single crystal X-ray diffraction** data of **1-A** were collected on a Nonius Kappa-CCD single crystal diffractometer using graphite mono-chromated Mo K_{α} radiation (λ = 0.71073 Å). A multi-scan absorption correction was performed. The structures were solved by direct methods using SHELXS-2014 and refined by full-matrix least squares on F^2 using SHELXL-2014.[1] Non-hydrogen atoms were refined anisotropically and hydrogen atoms were placed in calculated positions refined using idealized geometries (riding model) and assigned fixed isotropic displacement parameters. CCDC files 2171910, 2171931-2171934 contain the supplementary crystallographic data for this paper. These data can be obtained free of charge from The Cambridge Crystallographic Data Centre via www.ccdc.cam.ac.uk/data_request/cif. **Energy framework analysis** and **electrostatic potential** calculation were performed by using CrystalExplorer21.[2] Electrostatic potential and intermolecular interaction energies, which were partitioned into electrostatic (E_{elec}), polarization (E_{pol}), dispersion (E_{disp}) and repulsion (E_{rep}) energy components, were calculated based on the B3LYP/6-31 G(d,p) wave functions that were obtained by using the structural data from the corresponding CIF files. The obtained interaction energies were further utilized to map the network of energy frameworks across different pairs as cylindrical tubes joining the molecules. The radii of these cylinders are directly proportional to the strength of the corresponding intermolecular interactions.[3] **Visible images of macroscopic thin films of 1-C** were taken with a Nikon D5100 digital camera.

Table S1. Crystallographic data for **1-A**.

Temperature (K)	220
Empirical formula	$C_{36}H_{34}FeN_{12}O_4$
M_r	754.60
Crystal system	Orthorhombic
Space group	*Pbcn*
a (Å)	12.8503(4)
b (Å)	10.6078(3)
c (Å)	25.9297(11)
β (°)	90
V (Å^3)	3534.6(2)
Z	4
D_c (mg cm^{-3})	1.418
F(000)	1568
μ (Mo-K$_\alpha$) (mm^{-1})	0.49
No. of total reflections	24868
No. of reflections [$I > 2\sigma(I)$]	2159
R [$I > 2\sigma(I)$]	0.075
wR [$I > 2\sigma(I)$]	0.166
S	1.09

$R = \Sigma \,||F_o| - |F_c|| \,/\, \Sigma|F_o|$; $wR = [\,\Sigma\, [w(F_o^2 - F_c^2)^2] \,/\, \Sigma\, [w(F_o^2)^2]]^{1/2}$.
$w = 1/[\sigma^2(F_o^2) + (0.0385P)^2 + 3.9943P]$, where $P = (F_o^2 + 2F_c^2)/3$;

Table S2. Selected bond length (Å) and angles (°) of the title compounds.

**1-A** (LS)
Fe–N1 1.973(3)
Fe–N2 1.905(3)
Fe–N3 1.962(3)
N1—Fe—N1^{i} 90.98 (18)
N2^{i}—Fe—N1 96.35 (13)
N2—Fe—N1 79.79 (13)
N3^{i}—Fe—N1 92.21 (13)
N2i—Fe—N1^{i} 79.79 (13)
N2—Fe—N1^{i} 96.35 (13)
N3—Fe—N1^{i} 92.21 (13)
N2^{i}—Fe—N3^{i} 80.12 (14)
N2—Fe—N3^{i} 103.75 (13)
N2^{i}—Fe—N3 103.75 (14)
N2—Fe—N3 80.12 (14)
N3^{i}—Fe—N3 91.59 (19)

Symmetry code: (i) −*x*+1, *y*, −*z*+1/2

**1-B** (LS, 290 K)	**1-B** (HS, 300 K)	**1-C** (LS, 298 K)	**1-C** (HS, 298 K)
Fe–N1 1.979(15)	Fe–N1 2.140(9)	Fe–N1 1.97(3)	Fe–N1 2.23(2)
Fe–N3 1.908(11)	Fe–N3 2.149(8)	Fe–N1B 1.98(2)	Fe–N1B 2.21(2)
Fe–N4 1.970(5)	Fe–N4 2.125(9)	Fe–N3 1.90(4)	Fe–N3 2.10(2)
		Fe–N3B 1.91(4)	Fe–N3B 2.09(3)
N1–Fe–N3 80.7(3)	N1–Fe–N3 77.4(2)	Fe–N4 1.96(2)	Fe–N4 2.10(1)
N1–Fe–N1^{i} 94.3(6)	N1–Fe–N1^{i} 96.5(4)	Fe–N4B 1.96(2)	Fe–N4B 2.10(2)
N1–Fe–N3^{i} 99.8(4)	N1–Fe–N3^{i} 103.5(3)		
N1–Fe–N4 88.6(5)	N1–Fe–N4 88.8(3)	N1–Fe–N1B 87.7(8)	N1–Fe–N1B 81.4(6)
N3–Fe–N4 78.1(3)	N3–Fe–N4 77.11(19)	N1–Fe–N3 78.4(10)	N1–Fe–N3 77.3(7)
N1^{i}–Fe–N3 99.8(4)	N1^{i}–Fe–N3 103.5(3)	N1–Fe–N3B 98.0(10)	N1–Fe–N3B 91.1(9)
N3–Fe–N4^{i} 101.4(3)	N3–Fe–N4^{i} 102.0(2)	N1–Fe–N4B 94.1(9)	N1–Fe–N4B 102.6(6)
N1^{i}–Fe–N4 88.6(5)	N1^{i}–Fe–N4 88.8(3)	N1B–Fe–N3 89.2(9)	N1B–Fe–N3 82.4(7)
N3^{i}–Fe–N4 101.4(3)	N3^{i}–Fe–N4 102.0(2)	N1B–Fe–N3B 78.6(7)	N1B–Fe–N3B 77.5(7)
N4–Fe–N4^{i} 96.2(3)	N4–Fe–N4^{i} 97.1(4)	N1B–Fe–N4 98.1(7)	N1B–Fe–N4 98.4(6)
N1^{i}–Fe–N3^{i} 80.7(3)	N1^{i}–Fe–N3^{i} 77.4(2)	N3–Fe–N4 77.1(10)	N3–Fe–N4 77.2(7)
N3^{i}–Fe–N4 78.1(3)	N3^{i}–Fe–N4 77.11(19)	N3–Fe–N4B 111.0(9)	N3–Fe–N4B 123.3(7)
		N3B–Fe–N4 107.3(10)	N3B–Fe–N4 114.0(10)
Symmetry code: (i) −*x*+1, *y*, −*z*+1/2		N3B–Fe–N4B 81.2(7)	N3B–Fe–N4B 77.1(7)
		N4–Fe–N4B 88.8(8)	N4–Fe–N4B 88.7(5)

Table S3. Crystallographic data for **1-B** and **1-C**.

	**1-B**(LS)	**1-B**(HS)	**1-C**(LS)	**1-C**(HS)
Temperature (K)	290	300	298	298
Empirical formula	$C_{35.19}H_{26}FeN_{12}O_{3.19}$	$C_{35.60}H_{26}FeN_{12}O_{3.60}$	$C_{34}H_{26}FeN_{12}O_2$	
M_r	723.48	735.28	690.52	
Crystal system	orthorhombic		monoclinic	
Space group	*Pbcn*		$P2_1/c$	
a (Å)	13.0125(3)	13.1289(3)	13.3647(3)	13.1104(16)
b (Å)	10.0415(2)	9.8488(2)	21.1886(6)	19.4171(4)
c (Å)	24.6880(7)	25.1571(7)	11.4423(2)	12.7012(2)
β (°)			77.9356(9)	78.9023(7)
V (Å^3)	3225.86(17)	3252.90(15)	3168.65(13)	3172.75(10)
Z	4		4	
D_c (mg cm^{-3})	1.490	1.501	1.447	1.445
Wavelength (Å)	1.540596			
pd proc Is prof R factor	1.25	1.07	1.22	0.95
pd proc Is prof wR factor	1.60	1.41	1.89	1.37
pd proc Is prof wR expected	1.32	1.37	1.07	1.07
Refine Is goodness of fit all	1.20	1.03	1.76	1.28
Refine Is R factor all	0.46	0.59	1.51	1.19

Table S4. Short intermolecular contacts of **1-A** below the van der Waals radii.

**Contact**	**Length, Å**	**Symmetry operation of the contact atom**
N(6)···H(2O)	2.025(1)	x, y, z
H(3)···O(2)	2.357(1)	-1/2+x, -1/2+y, 1/2-z
H(5)···H(2O)	2.390(1)	-1/2+x, -1/2+y, 1/2-z
H(1)···N(5)	2.439(1)	1/2-x, -1/2+y, z
H(5)···O(2)	2.560(1)	-1/2+x, -1/2+y, 1/2-z
C(10)···H(17A)	2.634(1)	1/2-x, -1/2+y, z
N(5)···H(17A)	2.733(1)	1/2-x, -1/2+y, z
C(1)···H(7)	2.741(1)	1/2-x, -1/2+y, z
H(2)···C(15)	2.826(1)	1-x, -1+y, 1/2-z
N(6)···O(2)	2.839(1)	x, y, z
H(2)···C(14)	2.857(1)	1-x, -1+y, 1/2-z

Table S5. Short intermolecular contacts of **1-B** below the van der Waals radii.

Contact	Length, Å		Symmetry operation
	LS	HS	
C(1)···C(17)	3.044(30)	3.122(30)	x, 1-y, -1/2+z
C(1)···C(18)	2.819(30)	3.179(30)	x, 1+y, z
C(1)···H(17A)		2.888(30)	x, 1-y, -1/2+z
C(1)···H(17B)	2.896(30)		x, 1-y, -1/2+z
C(1)···H(17C)	2.665(30)	2.816(30)	x, 1-y, -1/2+z
C(10)···H(17B)	2.894(30)		1-x, 1-y, 1-z
C(13)···H(2)	2.799(30)		1-x, -1+y, 1/2-z
C(13)···O(2)		3.056(30)	1-x, y, 1/2-z
C(14)···H(2)	2.705(30)		1-x, -1+y, 1/2-z
C(15)···H(2)	2.781(30)	2.811(30)	1-x, -1+y, 1/2-z
C(15)···O(2)	2.979(30)		1/2-x, 1/2-y, 1/2+z
C(16)···H(2)		2.860(30)	1-x, -1+y, 1/2-z
C(17)···C(18)	2.925(30)		x, -y, 1/2+z
C(17)···O(2)		3.208(30)	1-x, y, 1/2-z
C(2)···C(17)	3.263(30)	3.330(30)	x, 1-y, -1/2+z
C(2)···H(17C)	2.579(30)	2.674(30)	x, 1-y, -1/2+z
C(7)···C(18)		3.288(30)	1/2+x, 1/2+y, 1/2-z
C(8)···C(18)		3.271(30)	1-x, 1+y, 1/2-z
C(9)···C(5)		3.326(30)	1.5-x, -1/2+y, z
H(1)···C(18)	1.951(30)	2.323(30)	x, 1+y, z
H(1)···H(7)	2.399(30)	2.305(30)	-1/2+x, 1/2+y, 1/2-z
H(14)···C(18)	2.615(30)	2.874(30)	x, -y, 1/2+z
H(15)···O(1)	2.516(30)	2.543(30)	-1/2+x, 1/2-y, 1-z
H(15)···O(2)	2.434(30)	2.445(30)	1/2-x, 1/2-y, 1/2+z
H(16)···C(3)	2.791(30)		-1/2+x, -1/2+y, 1/2-z
H(17A)···C(18)	2.363(30)	2.894(30)	x, -y, 1/2+z
H(17B)···C(18)	2.697(30)		x, -y, 1/2+z
H(7)···C(18)	2.762(30)	2.429(30)	1/2+x, 1/2+y, 1/2-z
H(7)···O(2)	2.700(30)		1/2+x, 1/2+y, 1/2-z
N(5)···C(18)		2.978(30)	1/2+x, 1/2+y, 1/2-z
N(5)···O(2)	2.951(30)		1/2+x, 1/2+y, 1/2-z
N(6)···H(3)	2.695(30)	2.510(30)	-1/2+x, -1/2+y, 1/2-z
N(6)···H(5)	2.575(30)	2.596(30)	-1/2+x, -1/2+y, 1/2-z
O(1)···O(2)	2.844(30)	2.276(30)	1-x, y, 1/2-z

Table S6. Short intermolecular contacts of **1-C** below the van der Waals radii.

**Contact**	**Length, Å**		**Symmetry operation of the contact atom**
	**LS**	**HS**	
C(1)···C(7b)	3.38(7)		x, 1/2-y, -1/2+z
C(1)···H(7b)	2.48(8)		x, 1/2-y, -1/2+z
C(13b)···H(14)	2.81(4)		-1+x, y, z
C(14b)···H(14)	2.78(9)	2.65(3)	-1+x, y, z
C(2)···H(7b)	2.71(7)	2.83(5)	x, 1/2-y, -1/2+z
C(3)···C(4)	3.36(8)		1-x, -y, -z
C(3)···N(6b)	3.09(9)		1-x, -y, -z
C(3b)···H(12)	2.68(9)	2.77(7)	x, 1/2-y, -1/2+z
C(2b)···H(17A)		2.83(3)	x, y, -1+z
C(3b)···H(17A)		2.88(4)	x, y, -1+z
C(3b)···H(17B)		2.76(3)	x, 1/2-y, -1/2+z
C(3b)···N(6)	3.08(8)		x, 1/2-y, -1/2+z
C(5b)···N(6)	3.18(7)		x, 1/2-y, -1/2+z
C(5b)···C(10b)		3.30(4)	x, 1/2-y, -1/2+z
C(6b)···C(10b)	3.38(8)	3.24(4)	x, 1/2-y, -1/2+z
C(6b)···C(11b)		3.17(5)	x, 1/2-y, -1/2+z
C(7b)···C(11b)		3.35(7)	x, 1/2-y, -1/2+z
C(7b)···C(12b)		3.31(6)	x, 1/2-y, -1/2+z
C(15b)···H(14)		2.59(5)	-1+x, y, z
C(16)···H(1b)		2.89(3)	2-x, -y, -z
H(16)···H(1b)	2.28(9)		2-x, -y, -z
H(17Cb)···O(1)	2.72(8)		-1+x, 1/2-y, 1/2+z
H(1)···H(12b)		2.30(3)	x, 1/2-y, -1/2+z
H(2)···C(13b)	2.83(7)		x, y, -1+z
H(2)···C(14b)	2.67(9)		x, y, -1+z
H(3)···N(6b)	2.15(8)	2.58(5)	1-x, -y, -z
H(2b)···C(17)		2.86(6)	x, y, -1+z
H(2b)···H(17A)		2.39(7)	x, y, -1+z
H(2b)···O(1)		2.66(6)	x, y, -1+z
H(3b)···H(12)	2.22(9)		x, 1/2-y, -1/2+z
H(3b)···N(6)	2.17(8)	2.41(6)	x, 1/2-y, -1/2+z
H(5b)···N(6)	2.27(7)	2.39(3)	x, 1/2-y, -1/2+z
H(6)···H(17Ab)	2.36(8)		1-x, -1/2+y, 1/2-z
H(16)···C(1b)		2.61(3)	2-x, -y, -z
H(16)···H(1b)		2.07(6)	2-x, -y, -z
H(17Bb)···C(13)		2.67(7)	-1+x, 1/2-y, 1/2+z
N(2b)···H(17B)		2.64(5)	x, 1/2-y, -1/2+z
N(5)···H(1b)		2.43(3)	2-x, -y, -z
N(5b)···H(17Cb)		2.69(3)	x, 1/2-y, -1/2+z

Table S7. The full color-coded interaction mappings of a central reference molecule with the nearest neighbors of **1-C** and the contributions to the total energy in both spin states as calculated by DTF-B3LYP/6-31G(d,p) method.

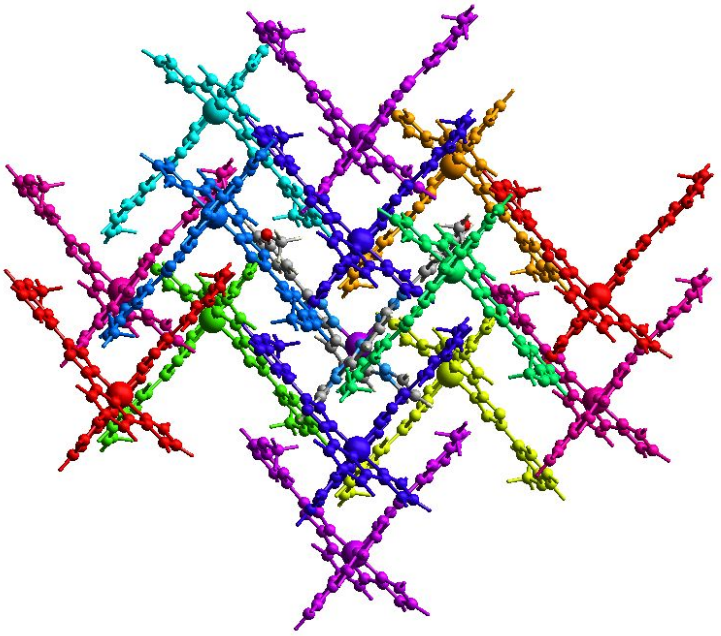

Color code	Symmetry operation	Spin state	*R*, Å	Δ*R*, Å	*E*, kJ mol^{-1}					
					Electrostatic	Polarization	Dispersion	Exchange-repulsion	**Total**	**Difference (LS–HS)**
	x, -*y*+1/2, *z*+1/2	LS	14.37	**-0.08**	-3.6	-1.4	-12.4	0.0	**-15.6**	**0.1**
		HS	14.29		-2.2	-1.7	-13.7	0.0	**-15.5**	
	-*x*, -*y*, -*z*	LS	10.01	**-0.11**	1.7	-6.4	-41.5	14.0	**-30.4**	**4.4**
		HS	9.90		7.5	-7.5	-44.3	16.6	**-26.0**	
	-*x*, -*y*, -*z*	LS	8.72	**0.61**	-91.3	-29.2	-100.2	120.4	**-131.1**	**18.5**
		HS	9.33		-60.4	-20.9	-74.1	50.6	**-112.6**	
	-*x*, -*y*, -*z*	LS	9.85	**-0.32**	-23.2	-9.0	-63.2	34.2	**-65.0**	**-37.6**
		HS	9.53		-73.5	-19.2	-72.8	85.4	**-102.6**	
	-*x*, *y*+1/2, -*z*+1/2	LS	11.78	**-0.67**	-6.1	-1.5	-13.6	7.7	**-14.7**	**-20.1**
		HS	11.11		-19.1	-4.7	-17.4	6.6	**-34.8**	
	-*x*, *y*+1/2, -*z*+1/2	LS	13.84	**-1.23**	-4.1	-0.7	-5.1	0.0	**-9.3**	**-17.0**
		HS	12.61		-12.5	-2.1	-13.3	0.0	**-26.3**	
	x, -*y*+1/2, *z*+1/2	LS	7.74	**0.28**	-71.3	-23.6	-117.7	143.1	**-106.9**	**-17.5**
		HS	8.02		-74.6	-27.5	-126.1	137.0	**-124.4**	
	x, *y*, *z*	LS	11.44	**1.26**	-19.0	-4.9	-32.6	24.3	**-37.0**	**-9.4**
		HS	12.70		-19.3	-4.1	-26.4	0.0	**-46.4**	
	x, *y*, *z*	LS	13.36	**-0.25**	8.7	-2.4	-20.9	0.0	**-10.8**	**1.2**
		HS	13.11		10.6	-3.4	-21.1	0.0	**-9.6**	
	-*x*, -*y*, -*z*	LS	13.66	**-**	10.2	-1.7	-8.7	0.0	**1.9**	**-1.9**
		HS	-		0	0	0	0	**0**	

Table S8. The full color-coded interaction mappings of a central reference molecule with the nearest neighbors of **1-B** and the contributions to the total energy in both spin states as calculated by DTF-B3LYP/6-31G(d,p) method. The methanol molecules were excluded from the calculations.

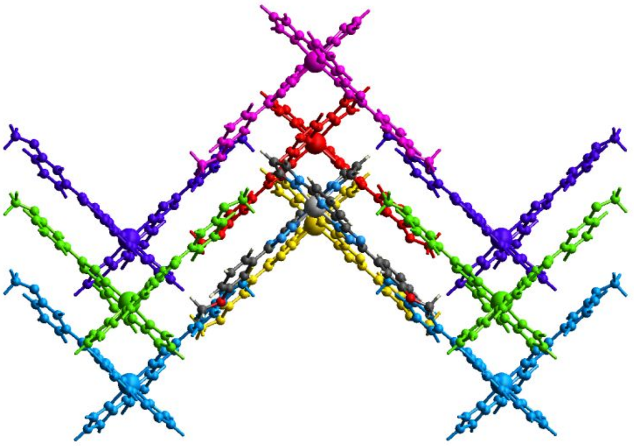

	**Symmetry operation**	**Spin state**	***R*, Å**	**Δ*R*, Å**	***E*, kJ mol^{-1}**					
					Electrostatic	Polarization	Dispersion	Exchange-repulsion	**Total**	**Difference (LS–HS)**
	x+1/2, *y*+1/2, -*z*+1/2	LS	8.22	**-0.01**	-27.5	-12.3	-72.4	53.0	**-68.5**	**-5.1**
		HS	8.21		-30.5	-16.8	-77.9	63.0	**-73.6**	
	x, y, z	LS	13.01	**0.10**	6.3	-1.0	-5.4	0.0	**1.2**	**0**
		HS	13.13		7.3	-1.3	-6.5	0.0	**1.2**	
	-*x*+1/2, -*y*+1/2, *z*+1/2	LS	16.30	**0.09**	-3.2	-1.7	-10.4	0.0	**-13.7**	**-3.6**
		HS	16.39		-6.2	-1.8	-10.9	0.0	**-17.3**	
	-*x*, -*y*, -*z*	LS	12.80	**0.02**	52.0	-10.6	-88.4	0.0	**-29.8**	**-6.0**
		HS	13.00		47.4	-13.6	-87.1	0.0	**-35.8**	
	-*x*, -*y*, -*z*	LS	18.25	**-0.07**	-0.4	-1.0	-10.9	0.0	**-10.7**	**3.2**
		HS	18.18		1.7	-1.2	-9.7	0.0	**-7.5**	
	x, *y*, *z*	LS	10.04	**-0.19**	-30.2	-6.6	-41.9	40.2	**-48.5**	**-3.5**
		HS	9.85		-28.7	-7.1	-36.9	25.5	**-52.0**	
	-*x*+1/2, -*y*+1/2, *z*+1/2	LS	-	**-**	0	0	0	0	**0**	**-4.5**
		HS	14.28		-0.5	-0.7	-3.9	0.0	**-4.5**	

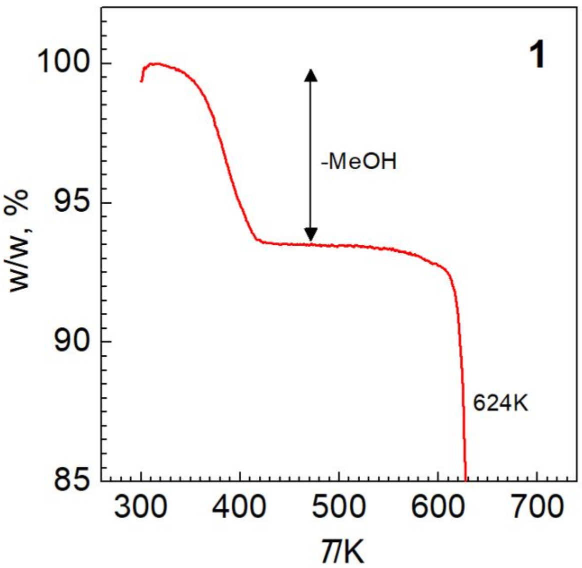

Figure S1. TGA profiles of the as-synthesized **1-A**.

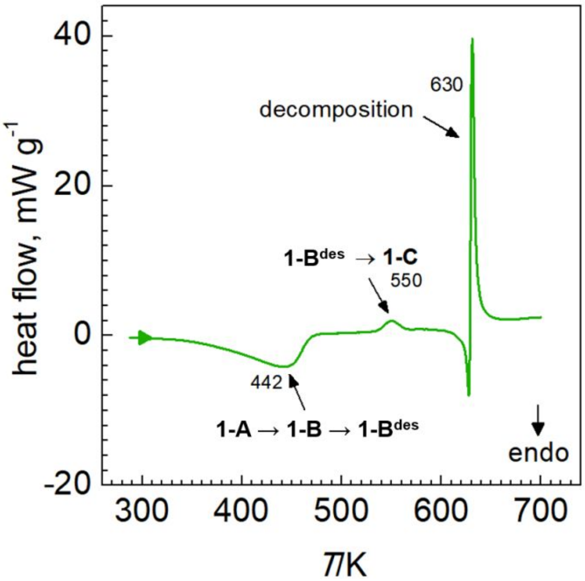

Figure S2. DCS profile of the as-synthesized **1-A**.

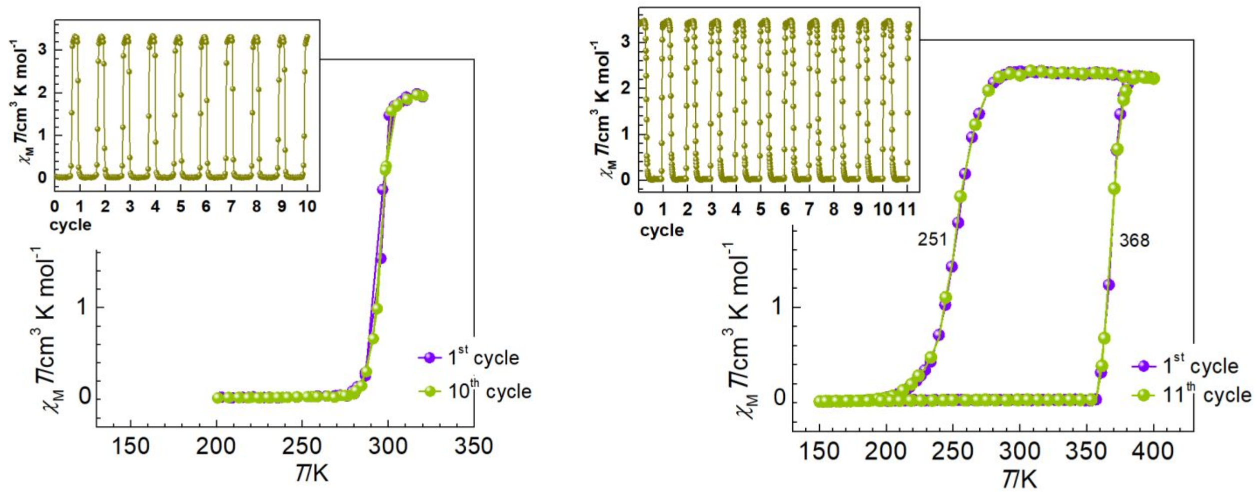

(a) (b)

Figure S3. Reproducibility of the ST behavior of **1-B** (a) and of **1-C** (b) at the rate 10 K min^{-1}. For the latter the hysteresis loop retains the shape observed at 0.1 K min^{-1} (Figure 1a), but expands to 116 K.

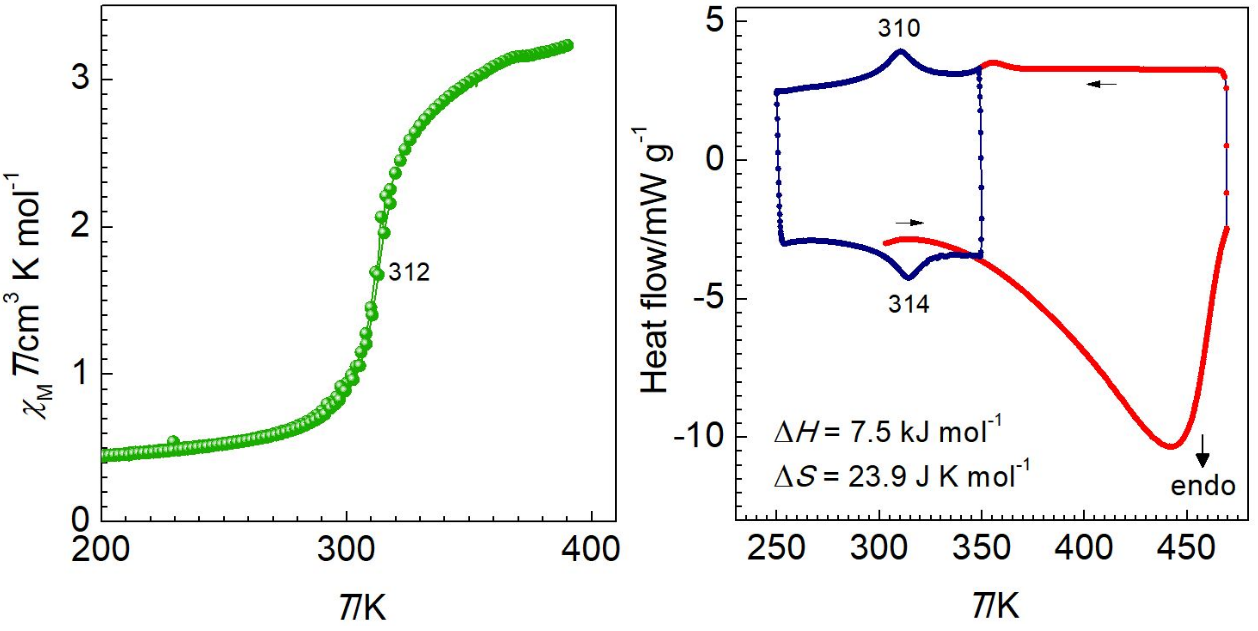

(a) (b)

Figure S4. (a) Magnetic curve of **1-B^{des}** at 1 K min^{-1}; (b) DSC profile of fresh **1-A**. The red line corresponds to the desolvation and polymorphic transformation to **1-B^{des}**, the dark blue line corresponds to the heating-cooling cycle of **1-B^{des}**.

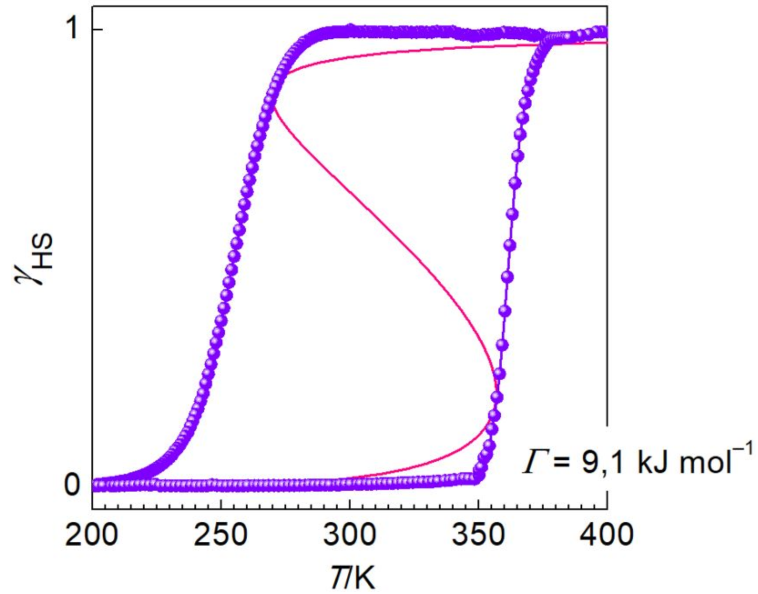

Figure S5. Fitting the magnetic curve of **1-C** by the Slichter-Drickamer model.

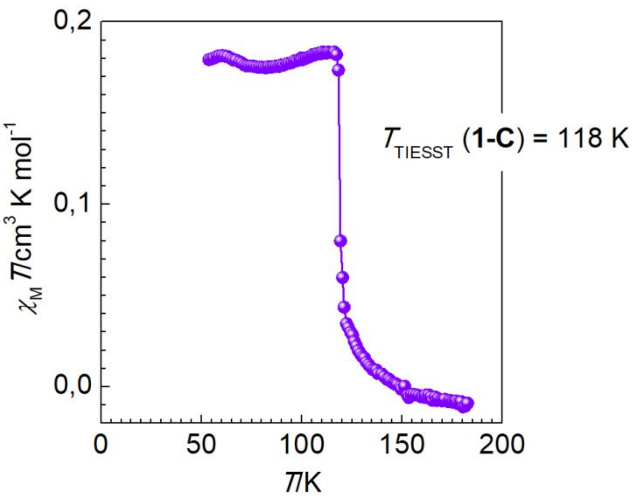

Figure S6. Relaxation TIESST curve of **1-C** at 0.3 K min^{-1}. Only *ca*. 6 % of the HS* molecules could be captured by rapid cooling inside the SQUID facility precooled at 10 K.

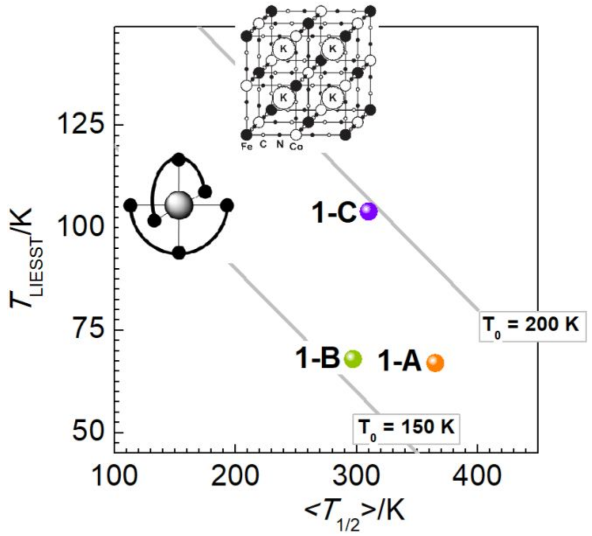

Figure S7. Comparison of the correlation T_{LIESST} vs. <$T_{1/2}$> for **1-A**, **1-B** and **1-C** with T_0 lines for tridentate based Fe^{II} complexes and polymeric CoFe Prussian blue analogues.[4-6]

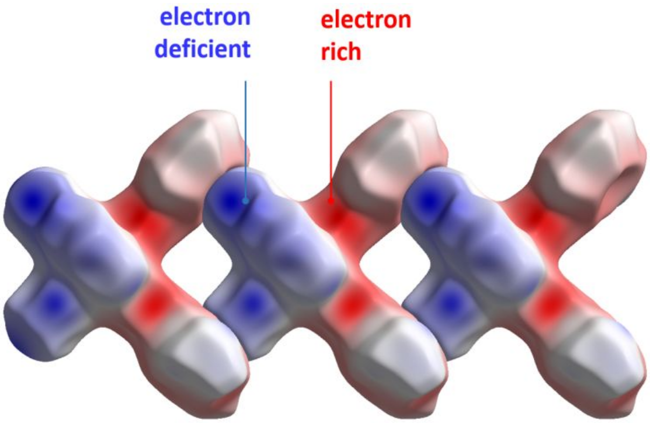

Figure S8. Electrostatic potential for the complex molecule of **1-A** derived from a B3LYP/6-31G(d,p) wavefunction mapped on the Hirshfeld surface in the range -0,1204 (red) to 0,1060 a.u. (blue). The negative charge is located on the triazole and phenyl moieties, while pyrazole and pyridine moieties are relatively positively charged.

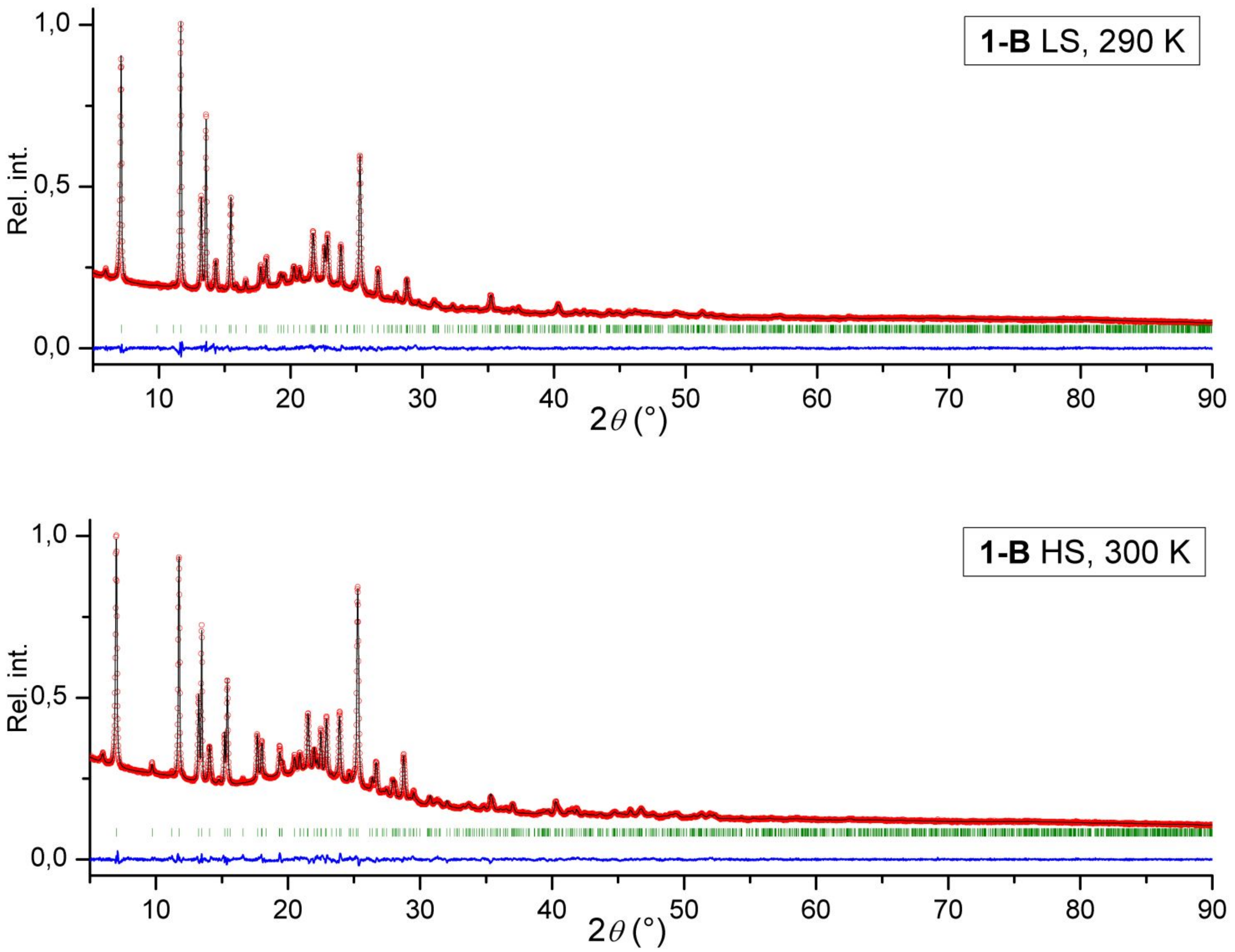

Figure S9. Rietveld refinement plots for **1-B** in both spin states at indicated temperatures. Red hollow dots and black solid line represent observed and calculated patterns, respectively, with peak markers and the difference plot shown at the bottom.

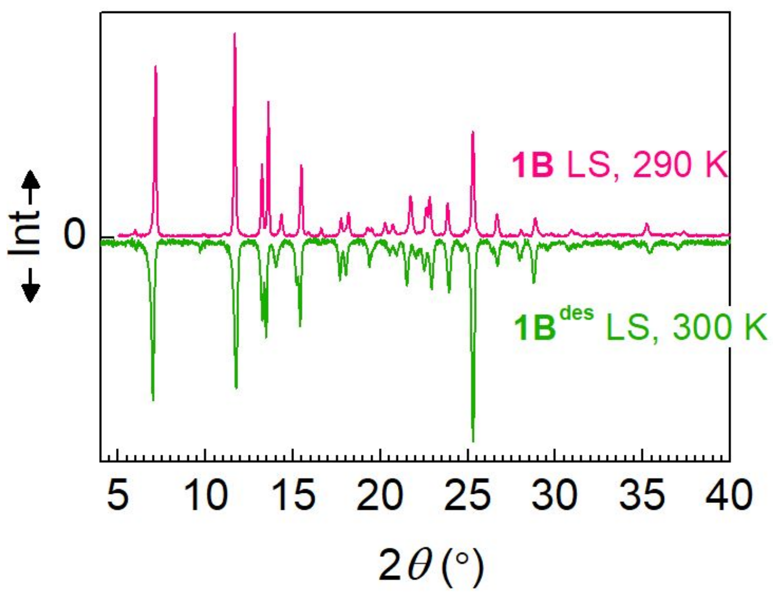

Figure S10. Comparison of the PXRD profiles of **1-B** and **1-B**^{des} in the LS state.

\

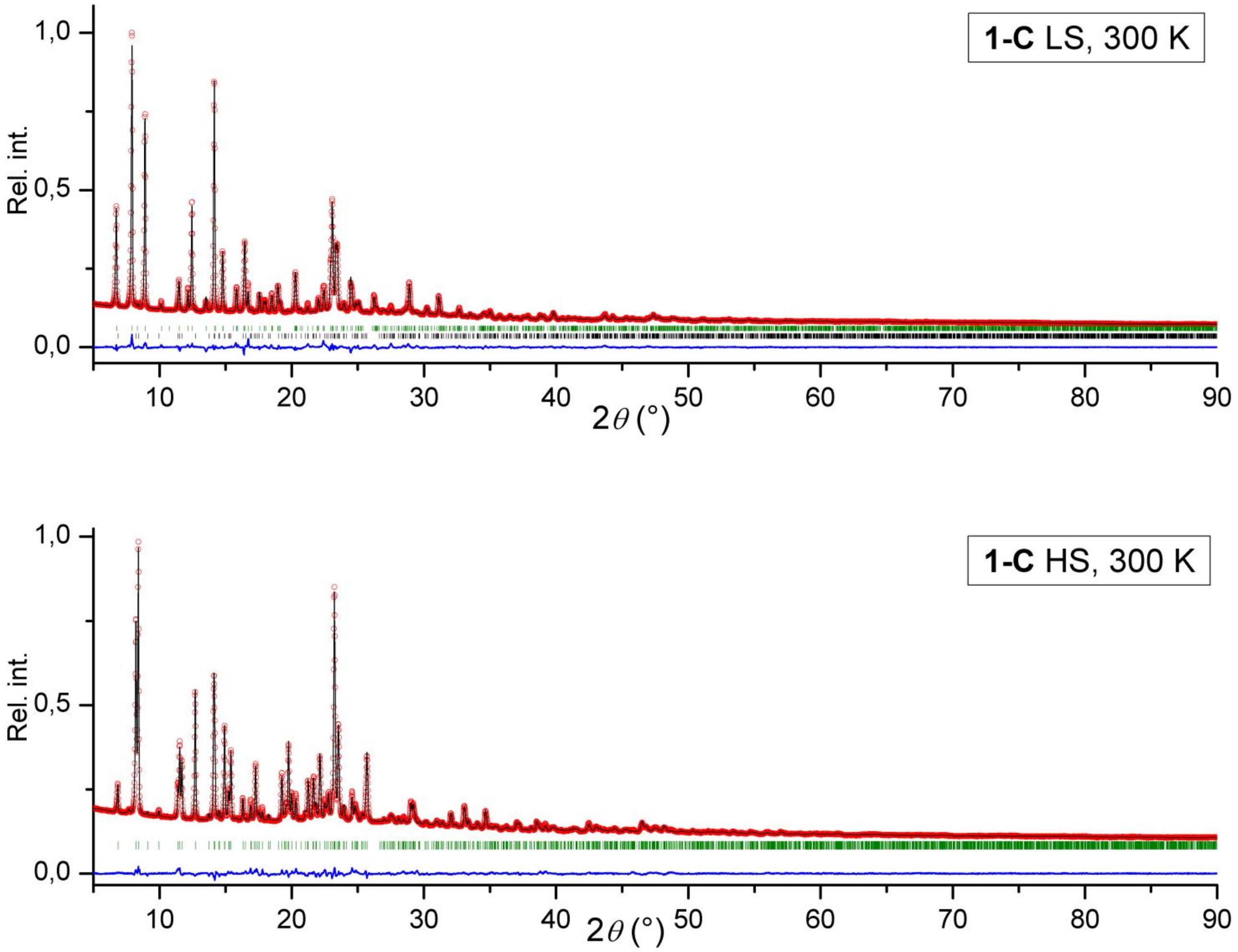

Figure S11. Rietveld refinement plots for **1-C** in both spin states at indicated temperature. Red hollow dots and black solid line represent observed and calculated patterns, respectively, with peak markers and the difference plot shown at the bottom. The LS phase has a 5% admixture of the HS phase, which was accounted for during the refinement process.

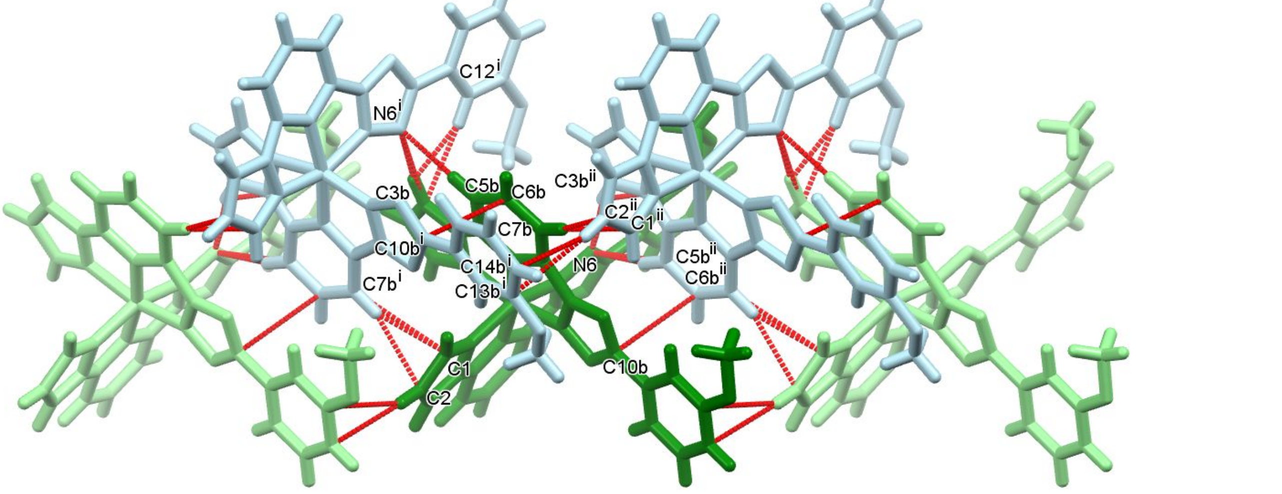

LS

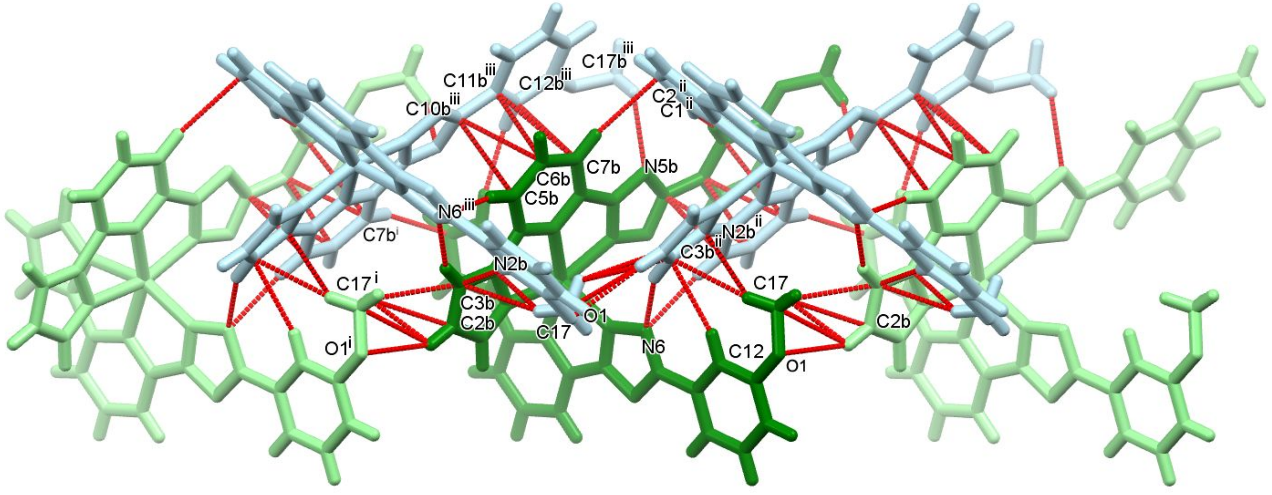

HS

Figure S12. The intermolecular contacts of a double chain of **1-C** in the LS and HS spin states. Note that the density of the intermolecular interactions below the vdW radii, shown as red dashed lines, increases in the HS state. Note, in the HS states the surrounding of the inverted complex molecule is shown for a better clarity.

LS **HS**

Coulomb energy:

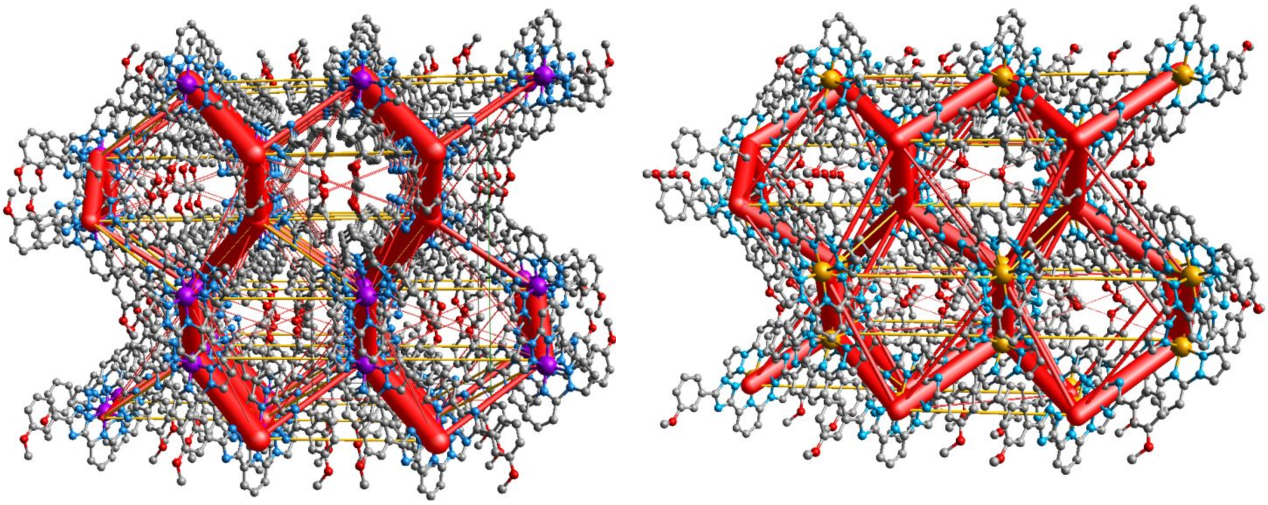

Dispersion energy:

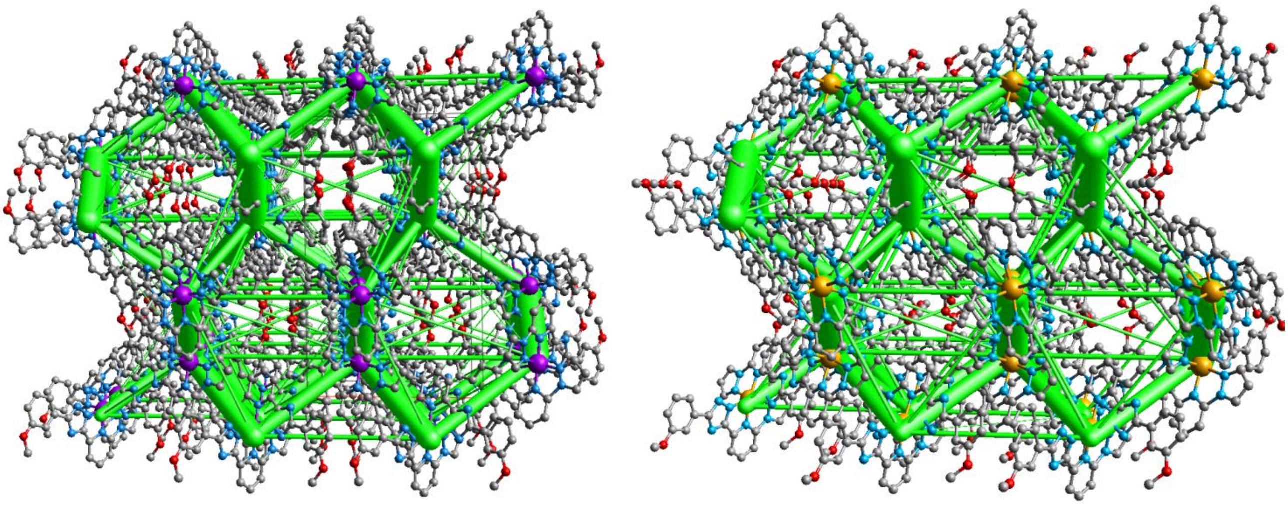

Total:

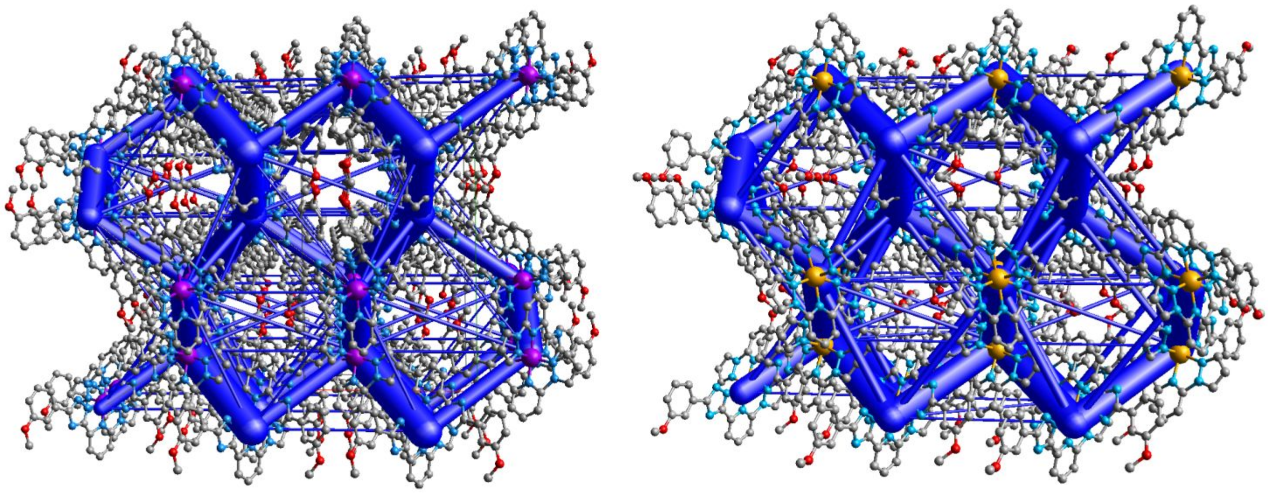

Figure S13. Coulomb and dispersion interaction energy components (above) of the total energy frameworks (below) of **1-C** in both spin states. The yellow cylinders correspond to repulsive interactions. Tube size is 100, cut-off is zero.

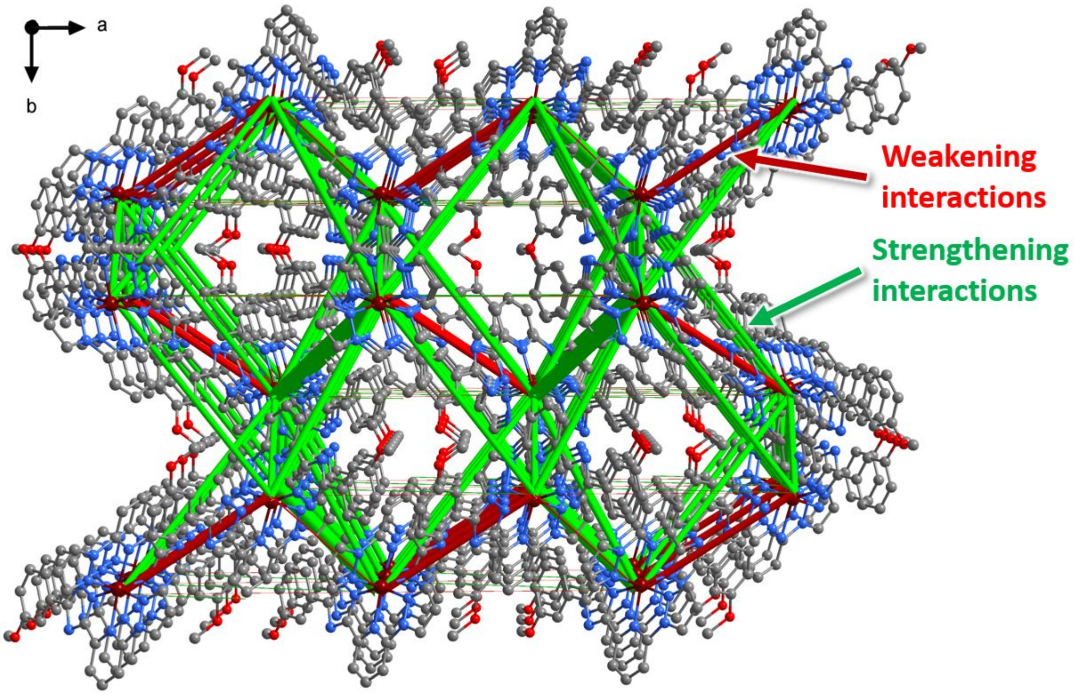

(a)

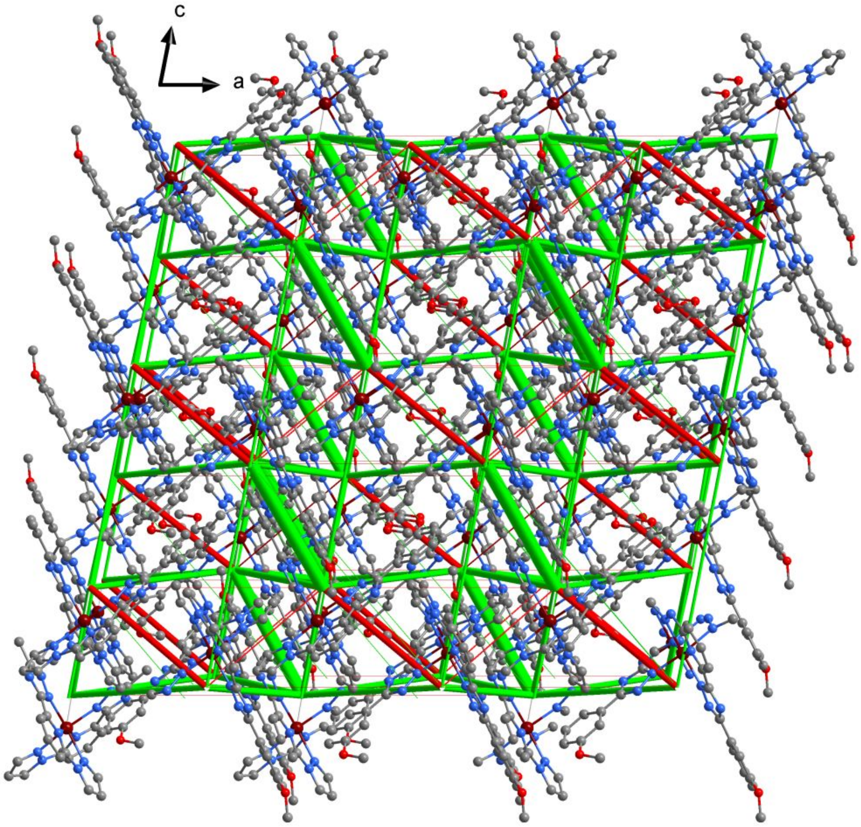

(b)

Figure S14. Differential framework of **1-C** constructed using values from the Table S7, column "Difference (LS–HS)", overlaid with a fragment of the crystal packing of the HS phase viewed along the *c* (a) and *b* (b) axes. The red cylinders correspond to weakening interactions, the green cylinders to the strengthening interactions. Tube size is scaled proportionally to the absolute value of the interaction energy, cut-off is zero.

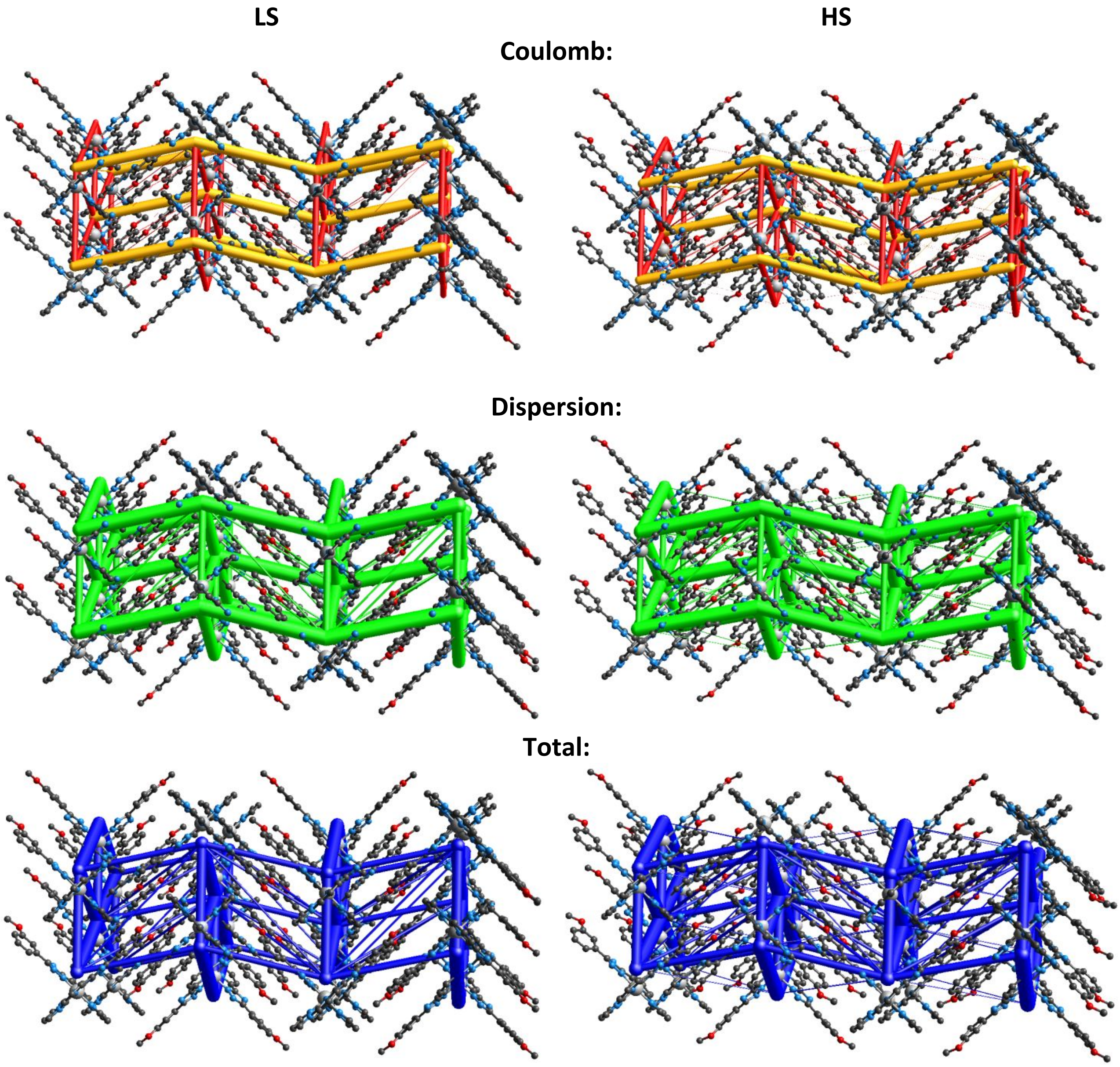

Figure S15. Coulomb and dispersion interaction energy components (above) of the total energy frameworks (below) of **1-B** in both spin states. The yellow cylinders correspond to repulsive interactions. Tube size is 100, cut-off is zero.

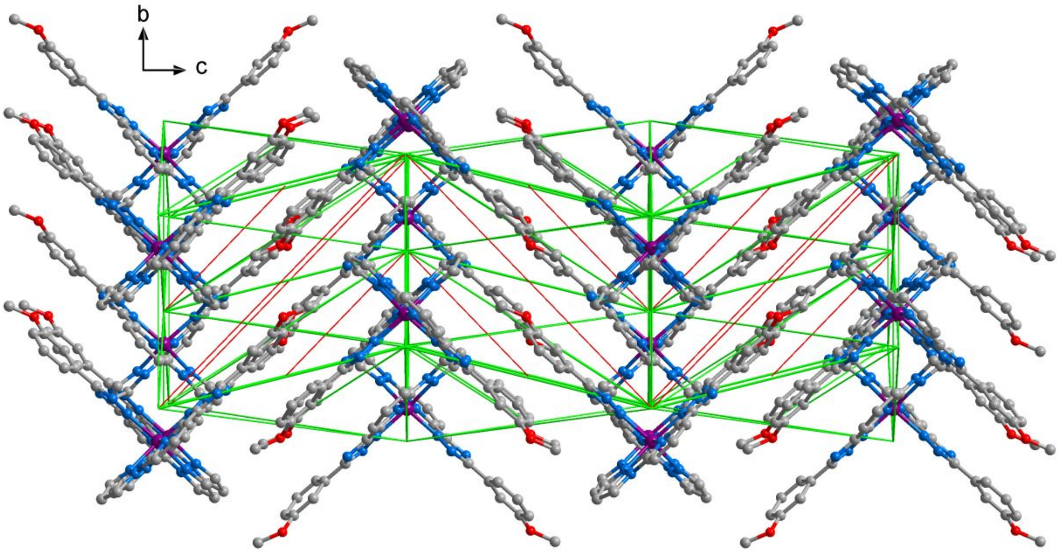

Figure S16. Differential framework of **1-B** constructed using values from the Table S8, column "Difference (LS–HS)", overlaid with a fragment of the crystal packing of the HS phase viewed along the *a* axis. The red cylinders correspond to weakening interactions, the green cylinders to the strengthening interactions. Tube size scale is the same as in Figure S14 and is proportional to the absolute value of the interaction energy. Cut-off is zero.

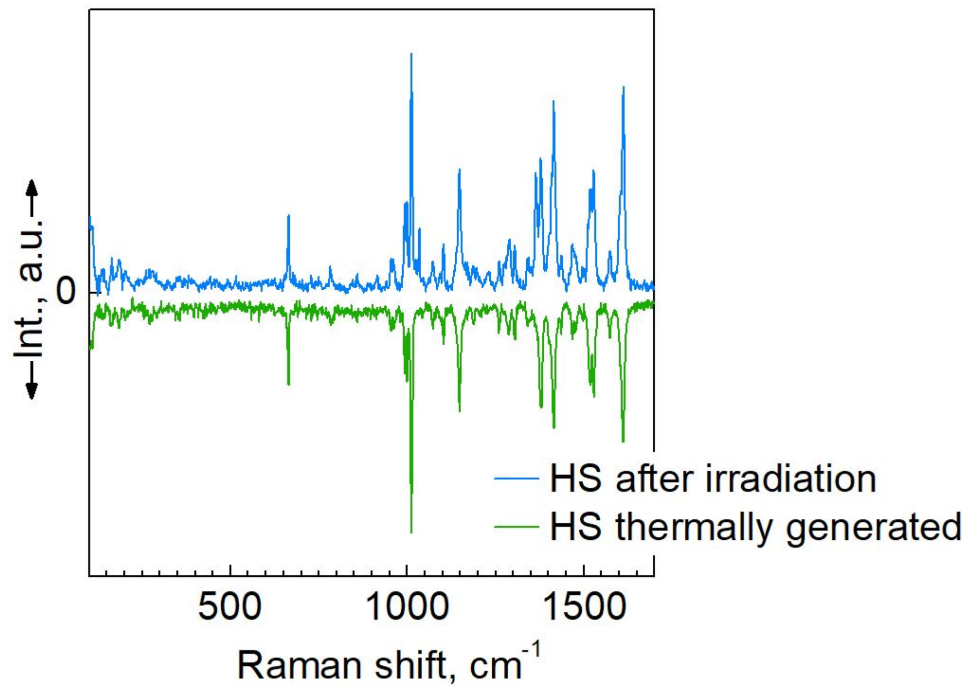

Figure S17. Comparison of Raman spectra of the HS phase of **1-C** generated thermally and by laser irradiation of the LS phase.

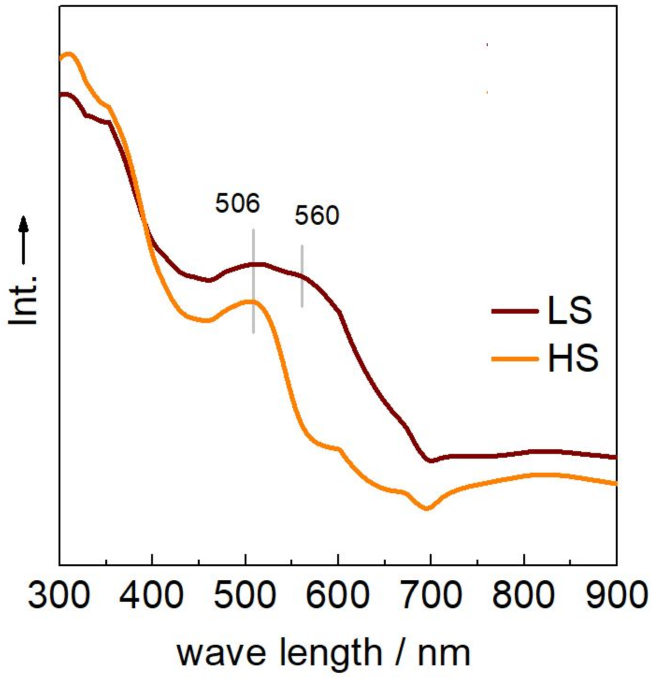

Figure S18. UV-vis spectra of the LS and the HS phases of **1-C** at RT.

Photoirradiation experiment with 1-C at RT

The formation of the image through light irradiation was done using a mask printed on a transparent adhesive film for laser printers (Lomond Copier Film).

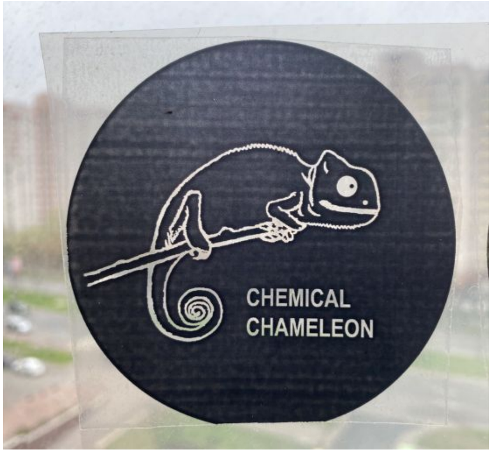

Irradiation of a microcrystalline sample of **1-C** in the LS state deposited as a thin film on filter paper leads to switching of areas under the mask without toner and the formation of the corresponding image.

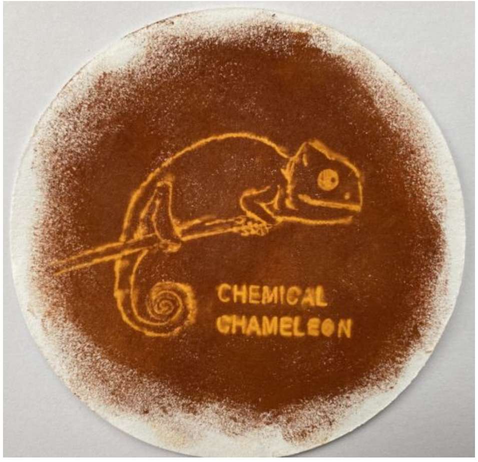

Alternatively, the film can be switched by moderate heating above the transition temperature with a heating gun:

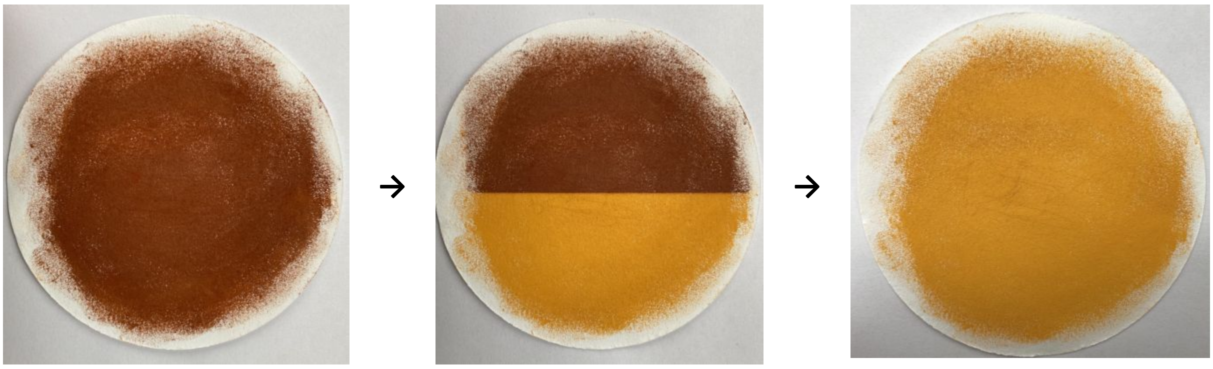

LS **LS+HS** **HS**

References

(1) Sheldrick, G. Crystal structure refinement with SHELXL. *Acta Crystallogr. Sect. C* **2015**, *71* (1), 3-8.

(2) Spackman, P. R.; Turner, M. J.; McKinnon, J. J.; Wolff, S. K.; Grimwood, D. J.; Jayatilaka, D.; Spackman, M. A. CrystalExplorer: a program for Hirshfeld surface analysis, visualization and quantitative analysis of molecular crystals. *J. Appl. Crystallogr.* **2021**, *54* (3), 1006-1011.

(3) Turner, M. J.; Thomas, S. P.; Shi, M. W.; Jayatilaka, D.; Spackman, M. A. Energy frameworks: insights into interaction anisotropy and the mechanical properties of molecular crystals. *Chem. Commun.* **2015**, *51* (18), 3735-3738.

(4) Létard, J. F. Photomagnetism of iron(II) spin crossover complexes - the T(LIESST) approach. *J. Mater. Chem.* **2006**, *16* (26), 2550-2559.

(5) Létard, J.-F.; Chastanet, G.; Guionneau, P.; Desplanches, C. In *Spin-Crossover Materials*; John Wiley & Sons Ltd: 2013, p 475-506.

(6) Chastanet, G.; Desplanches, C.; Baldé, C.; Rosa, P.; Marchivie, M.; Guionneau, P. A critical review of the T(LIESST) temperature in spin crossover materials − What it is and what it is not. *Chemistry Squared* **2018**, *2*, 2.
